# T cell senescence induced by DNMT1 deletion mediates cardiac transplant tolerance via altered CDKN1A methylation

**DOI:** 10.1016/j.ymthe.2026.02.015

**Published:** 2026-02-13

**Authors:** Yuqi Chen, Wai Yen Yim, Chenghao Li, Zihao Wang, Jincheng Hou, Yongbu Peng, Fayuan Liu, Zongtao Liu, Nianguo Dong, Yixuan Wang

**Affiliations:** 1 Department of Cardiovascular Surgery, Union Hospital, Tongji Medical College, Huazhong University of Science and Technology, Wuhan, China; 2 Department of Cardiovascular Surgery, Zhongnan Hospital of Wuhan University, Wuhan, China; 3 Key Laboratory of Organ Transplantation, Ministry of Education, NHC Key Laboratory of Organ Transplantation, Chinese Academy of Medical Sciences, Wuhan, China

**Keywords:** epigenetics, DNA methylation, T cell senescence, CDKN1A, cardiac transplantation

## Abstract

This study investigates the role of DNA methyltransferase 1 (DNMT1) in T cell senescence and heart transplant rejection using mouse models and clinical data. In the mouse heart transplantation model, DNMT1 was found to be highly expressed in graft-infiltrating T cells. Using *Dnmt1*^flox/flox^*Cd4*^cre/+^ mice, researchers demonstrated that T cell-specific knockout of DNMT1 led to long-term graft survival by impairing effector differentiation and promoting senescence in CD4^+^ T cells rather than in CD8^+^ T cells. Whole-genome methylation sequencing and RNA sequencing revealed that DNMT1 knockout decreased methylation of the cyclin-dependent kinase inhibitor 1A (CDKN1A) promoter, upregulating CDKN1A expression. Depletion of CDKN1A^high^ cells reversed DNMT1-inhibitor-induced T cell senescence. Clinically, increased T cell senescence was observed in post-transplant patients and is known to increase with age. Additionally, data from the UNOS database showed lower rejection rates in older recipients (age >65). These findings suggest that DNMT1 ablation accelerates CD4^+^ T cell senescence by altering CDKN1A methylation, thereby reducing T cell function and mitigating rejection. DNMT1 may thus serve as a potential therapeutic target for heart transplant rejection.

## Graphical abstract

**Figure:**
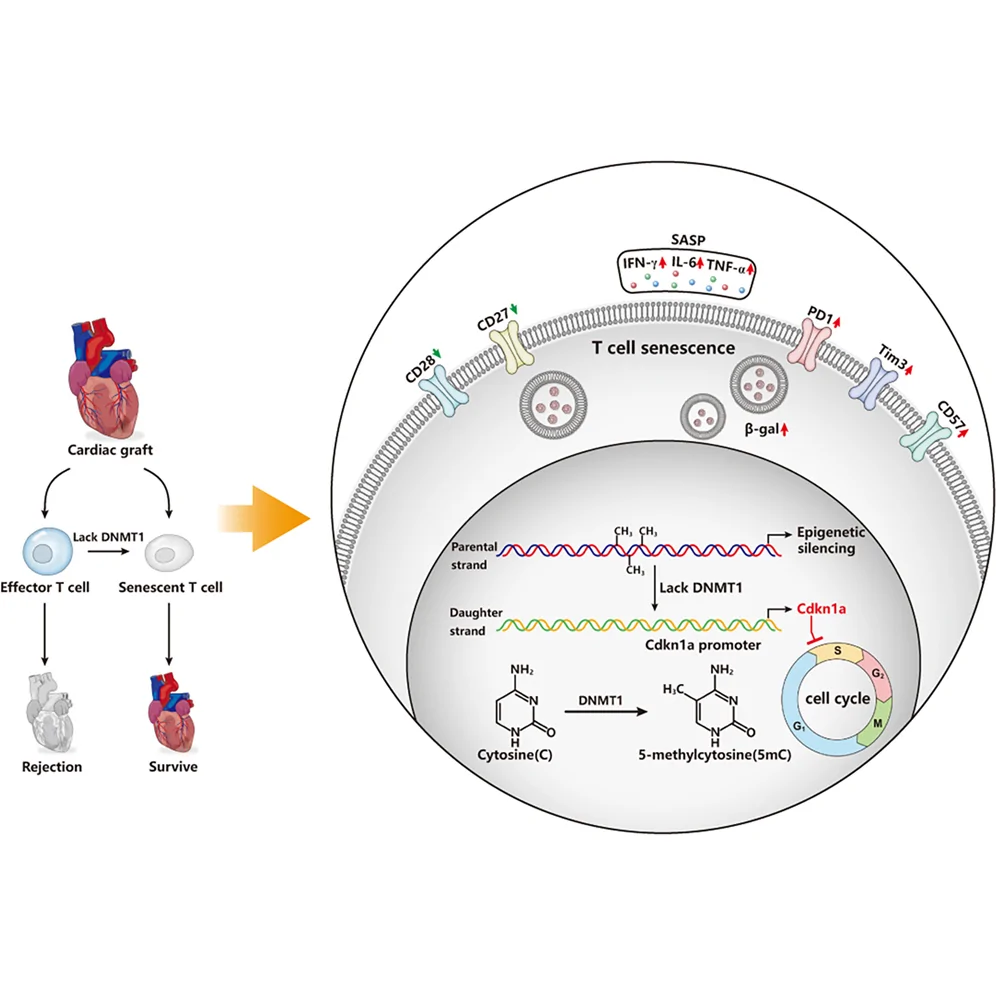

## Teaser

DNMT1 loss in CD4^+^ T cells accelerates senescence via CDKN1A hypomethylation, enabling long-term cardiac graft survival.

## Introduction

Cardiac transplantation is the most effective treatment for end-stage heart failure, and rejection reactions are the main challenge affecting the survival of transplantation.^1^ T cells, which are rapidly activated and produce effector functions upon stimulation by allogeneic antigens, play a central role in transplant rejection reactions.^2^ Additionally, under the influence of individual aging and various internal and external stimuli, T cells undergo senescence. Senescent T cells reduce or lose their ability to respond to allogeneic antigens and fail to exert effector functions.^3^ Targeting T cell effector differentiation and intervening in T cell functional changes to find an effective target for drug treatment is one of the main directions in combating transplant rejection reactions.

The inheritable changes in gene expression that occur during mitosis or meiosis without altering the genetic sequence are called epigenetics.^4^ DNA methylation is an important aspect of epigenetics, affecting gene accessibility and regulating gene expression by altering the methylation status at the fifth carbon position of cytosine C.^5^ DNA methylation plays a key role in the maturation process of T cells in the thymus.^6^ The differentiation direction and gene expression of secretory factors in CD4^+^ T cells are also closely related to DNA methylation patterns.^7^ In cytotoxic CD8^+^ T cells, the methylation enrichment regions of differential transcription factors have been proven to be key to their cytotoxic effects.^8^ DNMT1, a core molecule of DNA methylation, has been shown in numerous studies to regulate the expression levels of Foxp3, a key functional molecule in regulatory T cells (Tregs), and its activity has been recognized as a key factor affecting T cell reactivity in autoimmune diseases.^9^^,^^10^ These studies indicate that DNMT1 is a key factor in the epigenetic regulation of key genes in T lymphocytes, affecting T cell function. However, in the field of transplantation, whether DNA methylation and DNMT1 in T cells are related to rejection reactions requires further research.

To investigate the role of DNMT1 in T cell-mediated cardiac transplant rejection, we generated a strain of *Dnmt1*^flox/flox^ *Cd4*^cre/+^ mice in which DNMT1 expression is lost following the activation of Cre recombinase under the control of the CD4 promoter, thereby specifically deleting DNMT1 in T cells. Our study found that, in a cardiac transplant model, the formation of effector T cells was impaired in *Dnmt1*^flox/flox^ *Cd4*^cre/+^ mice, while the number of senescent T cells increased, leading to an impairment in T cell proliferation and effector functions, which resulted in long-term graft survival. In subsequent studies, we found that the methylation of the promoter of the key gene CDKN1A, associated with cellular senescence, was reduced in T cells of *Dnmt1*^flox/flox^ *Cd4*^cre/+^ mice, and the expression of the protein CDKN1A was increased. Depletion of cells expressing CDKN1A can reverse the T cell phenotype induced by the DNMT1 inhibitor 5-Azac (5-azacytidine).

## Results

### T cell-specific knockout of DNMT1 can lead to long-term graft survival

**Figure 1:**
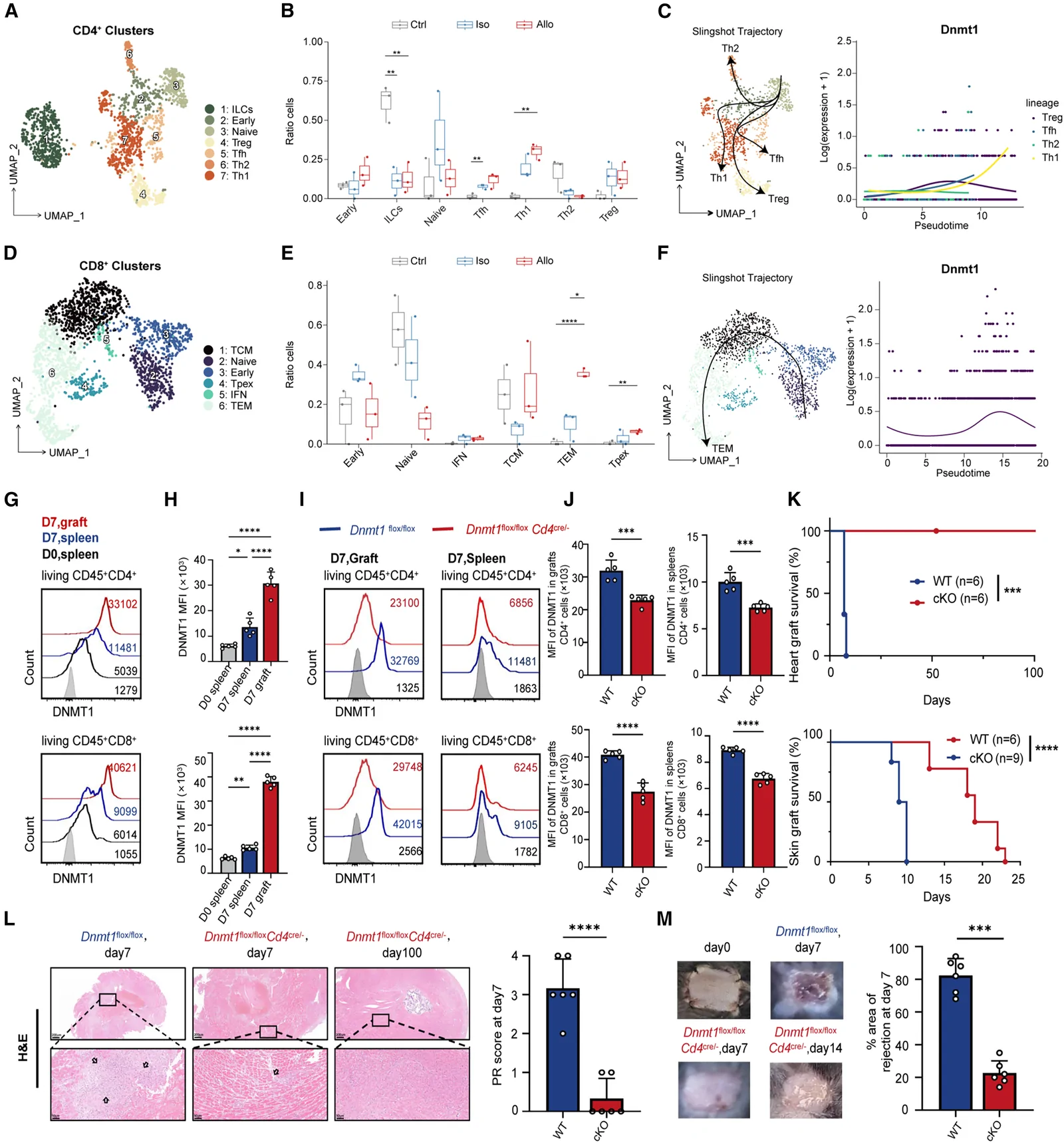
T cell-specific knockout of DNMT1 can lead to long-term graft survival (A) UMAP plot of CD4^+^ T cells from nine samples across three experimental groups (control, isograft, and allograft). Cells were colored by their computationally assigned state, revealing seven distinct clusters: innate lymphoid cells, early activation, naive, regulatory T cells (Treg), T follicular helper (Tfh) cells, T helper 2 (Th2) cells, and T helper 1 (Th1) cells. (B) Proportional abundance of each CD4^+^ T cell state across the three groups. Each of the nine dots corresponds to one independent biological sample (*n* = 3 for Ctrl, *n* = 3 for isograft, and *n* = 3 for allograft). Boxplots show the median and interquartile range. Statistical significance was determined by a non-parametric Wilcoxon rank-sum test (∗*p* < 0.05 and ∗∗*p* < 0.01). (C) Pseudotemporal trajectory analysis (Slingshot) inferring four distinct CD4^+^ T cell differentiation paths from a naive state: (1) naive to Treg, (2) naive to Tfh, (3) naive to Th2, and (4) naive to Th1 (left). The expression dynamics of Dnmt1 along the four inferred trajectories with a smoothed regression line showing the trend of Dnmt1 expression change (log-normalized counts) over pseudotime for each trajectory (right). (D) UMAP plot of CD8^+^ T cells colored by their computationally assigned state, revealing seven distinct clusters: central memory T (TCM), naive, early activation, progenitor exhausted T (Tpex) cells, interferon stimulated (IFN), and effector memory T (TEM). (E) Proportional abundance of each CD8^+^ T cell state across the three experimental groups. Each of the nine dots corresponds to one independent biological sample (*n* = 3 for Ctrl, *n* = 3 for isograft, and *n* = 3 for allograft). Boxplots show the median and interquartile range. Statistical significance was determined by a non-parametric Wilcoxon rank-sum test (∗*p* < 0.05, ∗∗*p* < 0.01, ∗∗∗*p* < 0.005, and ∗∗∗∗*p* < 0.001) (F) Pseudotemporal trajectory analysis (Slingshot) inferring naive to TEM state (left). Expression dynamics of Dnmt1 along the inferred trajectory with a smoothed regression line showing the trend of Dnmt1 expression change (log-normalized counts) over pseudotime for each trajectory (right). (G and H) Representative histogram overlay and median fluorescence intensity (MFI) bar graphs of DNMT1 from spleen and graft CD4^+^ and CD8^+^ T cells at days 0 and 7 after cardiac transplantation. Numbers on the right indicate MFI values for respective colored samples. ∗*p* < 0.05, ∗∗*p* < 0.01, and ∗∗∗∗*p* < 0.0001; multiple comparisons following ANOVA. (I and J) Representative histogram overlay and MFI bar graphs of DNMT1 from spleen and graft CD4^+^ and CD8^+^ T cells of *Dnmt1*^flox/flox^*Cd4*^cre/+^ and *Dnmt1*^flox/flox^ control mice at day 7 after cardiac transplantation. Numbers on the right indicate MFI values for respective colored samples. ∗∗∗*p* < 0.001 and ∗∗∗∗*p* < 0.0001; unpaired Student’s *t* test. (K) Percentage of heart (*n* = 6) and skin (WT *n* = 6 and conditional knockout, cKO *n* = 9) allograft survival after transplantation. ∗∗∗*p* < 0.001 and ∗∗∗∗*p* < 0.0001; log rank test. (L) Representative images of H&E staining of grafts from *Dnmt1*^flox/flox^*Cd4*^cre/+^ and control mice after cardiac transplantation. Original magnification, ×50 and ×200. Bar graphs of PR score at day 7 after cardiac transplantation. *n* = 6, ∗∗∗*p* < 0.001; unpaired Student’s *t* test. (M) Representative images of rejected and accepted tail skin allografts after transplantation and bar graphs of percentage rejection area at day 7 of *Dnmt1*^flox/flox^*Cd4*^cre/+^ and control mice after transplantation. *n* = 6, ∗∗∗*p* < 0.001; unpaired Student’s *t* test. All results are expressed as the mean ± standard deviation, with *n* = 5 unless otherwise specified.

To define the immune mechanisms underlying the long-term graft survival conferred by T cell-specific DNA methylation, we performed an integrated single-cell RNA sequencing (scRNA-seq) analysis of cardiac allografts. We first profiled the global immune landscape, integrating data from nine samples across control, syngeneic (iso-graft), and allogeneic (allo-graft) groups (Figure S1A). This analysis identified all major immune lineages—myeloid cells, B cells, neutrophils, dendritic cells (DCs), natural killer (NK) cells, and T cells—which were validated by canonical marker genes (Figure S1B). Although derived from different datasets, the data demonstrated good consistency upon analysis (Figure S2).Quantification of cellular abundance revealed shifts in the immune compartment during rejection, with increasing T cells in allografts (Figure S1C). We next performed a deep characterization of the T cell compartment, beginning with CD4^+^ T cells. Unsupervised clustering revealed seven distinct states, including naive, Treg, T follicular helper (Tfh), T helper 2 (Th2), Th1, early activation, and innate lymphoid cells (ILCs) (Figures 1A and S1D). Comparative analysis demonstrated that allograft rejection was characterized by a significant remodeling of the CD4^+^ T cell state, marked by a pronounced expansion of Th1 cells and a concomitant reduction in naive cells (Figure 1B). The exact *p* values and effect sizes are shown in Table S1. This shift was functionally consequential, as Th1 cells exhibited significantly elevated scores for “activation/effector,” “adhesion,” “cytotoxicity,” “chemokine,” and “TCR signaling” gene programs (Figure S1E). Intriguingly, differential expression analysis identified canonical effectors such as *Il12rb2*, *Tbx21*, and *Ifng* in the pathogenic Th1 population alongside the DNA methyltransferase *Dnmt1* (Figure S1F). Pseudotemporal trajectory analysis showed four distinct differentiation paths from a naive state, and crucially, *Dnmt1* expression increased sharply and specifically along the trajectory culminating in Th1 cells (Figure 1C).

We observed a parallel phenomenon in the CD8^+^ T cell lineage. Clustering analysis defined six states, including naive, central memory (TCM), early activation, progenitor exhausted (Tpex), effector memory (TEM), and an interferon (IFN)-stimulated population (Figures 1D and S1G). In allografts, we documented a dramatic shift toward terminal effector differentiation, evidenced by a massive expansion of TEM cells (Figure 1E). These allograft-derived TEM cells similarly displayed enhanced “activation/effector” and “cytotoxicity” programs (Figure S1H). Mirroring our CD4^+^ findings, *Dnmt1* was significantly upregulated in CD8^+^ TEM cells (Figure S1I), and its expression increased steadily along the developmental trajectory from naive to TEM (Figure 1F).

To gain mechanistic insight into these differentiation processes, we performed Mfuzz clustering of genes dynamically expressed along the naïve-to-Th1 and naïve-to-TEM trajectories. This analysis revealed co-expression modules with distinct temporal patterns (Figures S1J and S1K). Gene Ontology analysis indicated that these modules were enriched for critical biological processes, including T cell activation, cytokine production, and regulation of immune response, effectively charting the transcriptional programs driving effector differentiation. Finally, we confirmed that the high Dnmt1 expression observed in Th1 and TEM cells was specific to the allograft environment (Figure S1L) and was functionally coupled to the heightened “activation/Effector” and “cytotoxicity” scores^11^ that define these populations (Figures S1M and S1N).

To validate the findings from the single-cell analysis, the mouse heart transplantation model was established. The expression of DNMT1 in both CD4^+^ and CD8^+^ T cells was measured by flow cytometry and found to be upregulated during acute rejection reactions in the mouse heart transplant model (Figures 1G and 1H). Additionally, the expression of DNMT1 was observed to be significantly positively correlated with T cell-mediated rejection reactions by analyzing the sequencing results from endomyocardial biopsy specimens of patients with the acute rejection reactions (Figure S3A). The increase in DNMT1 was also observed in T cells activated *in vitro* with CD3 and anti-CD28 stimulation (Figures S3B and S3C). These findings suggested that DNMT1 may be involved in the activation process of T cells during rejection reactions.

To investigate the impact of DNMT1 on heart transplant rejection, we constructed *Dnmt1*^flox/flox^ *Cd4*^cre/+^ mice, whose T cells were specifically knocked out for DNMT1 (Figures 1I and 1J). The mice had relatively normal counts of T cells in the peripheral immune organs under immunologically quiescent conditions (Figure S4). But lack of DNMT1 in T cells significantly prolonged the survival time of heart grafts (male mice were monitored for >100 days, whereas female mice were monitored for >50 days) and skin grafts (median survival time was 23 days compared with the *Dnmt1*^flox/flox^ control group’s 9 days) (Figures 1K and S5A). On day 7 after heart transplantation, the infiltration of inflammatory cells in the graft hearts of *Dnmt1*^flox/flox^ *Cd4*^cre/+^ mice was reduced, and the parenchyma rejection (PR) score was decreased (Figures 1L, S5B, and S5C). On day 7 after skin transplantation, the area of rejection in the skin grafts of *Dnmt1*^flox/flox^ *Cd4*^cre/+^ mice was smaller than that of the control group (Figure 1M).

### T cell-specific knockout of DNMT1 can lead to impaired effector differentiation of T cells in heart transplantation

**Figure 2:**
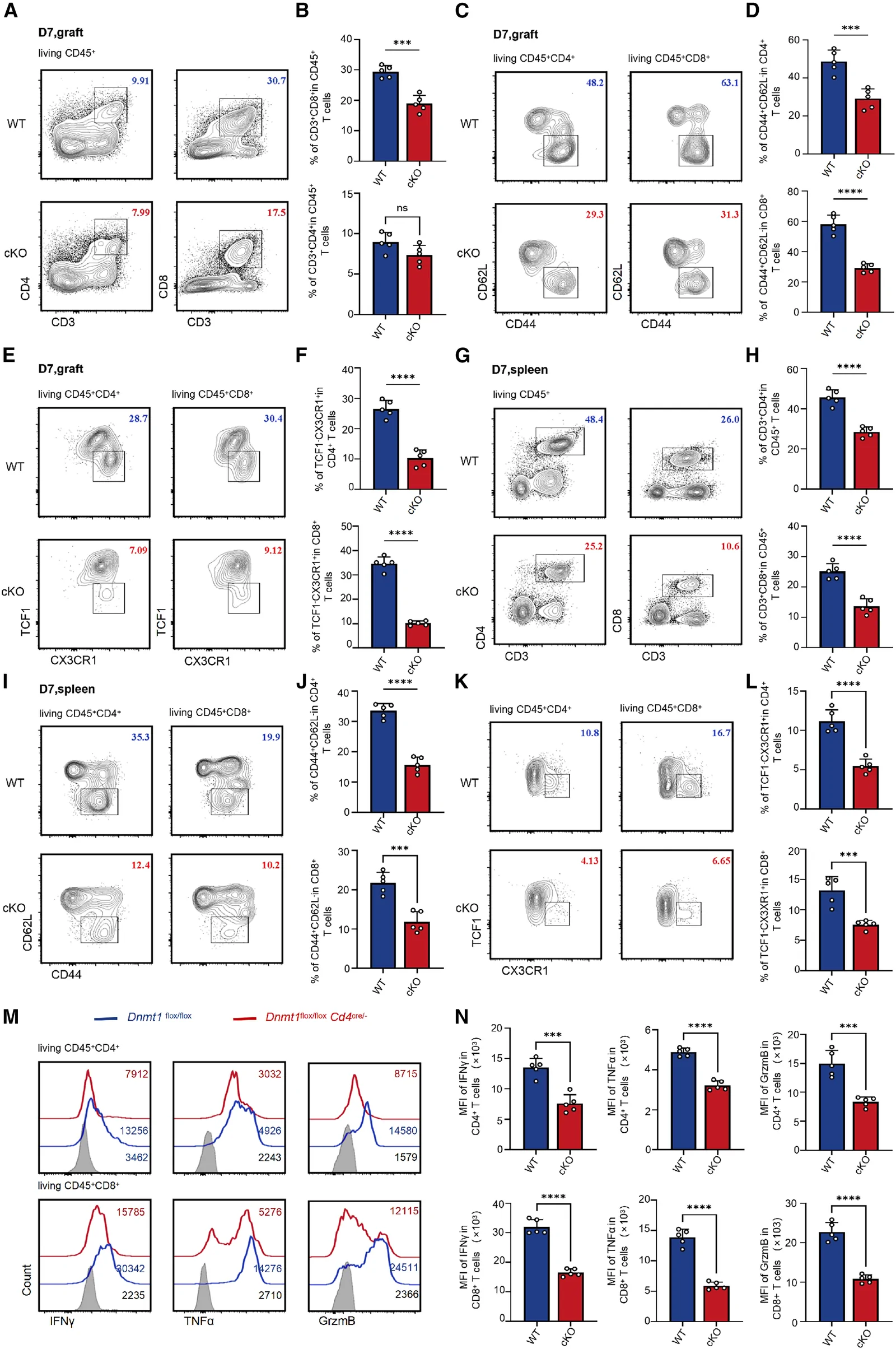
T cell-specific knockout of DNMT1 can lead to impaired effector differentiation of T cells in heart transplantation (A and B) Representative contour plots and bar graphs showing the percentage of CD4^+^ and CD8^+^ T cells in CD45^+^ cells of grafts from *Dnmt1*^flox/flox^*Cd4*^cre/+^ and *Dnmt1*^flox/flox^ control mice at day 7 after cardiac transplantation. ns, no significance; ∗∗∗*p* < 0.001; unpaired Student’s *t* test. (C and D) Representative contour plots and bar graphs showing the percentage of CD44^+^CD62L^−^ cells in CD4^+^ and CD8^+^ T cells of grafts from *Dnmt1*^flox/flox^*Cd4*^cre/+^ and *Dnmt1*^flox/flox^ control mice at day 7 after cardiac transplantation. ∗∗∗*p* < 0.001 and ∗∗∗∗*p* < 0.0001; unpaired Student’s *t* test. (E and F) Representative contour plots and bar graphs showing the percentage of CX3CR1^+^TCF1^−^ T cells in CD4^+^ and CD8^+^ T cells of grafts from *Dnmt1*^flox/flox^*Cd4*^cre/+^ and *Dnmt1*^flox/flox^ control mice at day 7 after cardiac transplantation. ∗∗∗∗*p* < 0.0001; unpaired Student’s *t* test. (G and H) Representative contour plots and bar graphs showing the percentage of CD4^+^ and CD8^+^ T cells in CD45^+^ cells of spleens from *Dnmt1*^flox/flox^*Cd4*^cre/+^ and *Dnmt1*^flox/flox^ control mice at day 7 after cardiac transplantation. ∗∗∗*p* < 0.001 and ∗∗∗∗*p* < 0.0001; unpaired Student’s *t* test. (I and J) Representative contour plots and bar graphs showing the percentage of CD44^+^CD62L^−^ T cells in CD4^+^ and CD8^+^ T cells of spleens from *Dnmt1*^flox/flox^*Cd4*^cre/+^ and *Dnmt1*^flox/flox^ control mice at day 7 after cardiac transplantation. ∗∗∗∗*p* < 0.0001; unpaired Student’s *t* test. (K and L) Representative contour plots and bar graphs showing the percentage of CX3CR1^+^TCF1^−^ T cells in CD4^+^ and CD8^+^ T cells of spleens from *Dnmt1*^flox/flox^*Cd4*^cre/+^ and *Dnmt1*^flox/flox^ control mice at day 7 after cardiac transplantation. ∗∗∗*p* < 0.001 and ∗∗∗∗*p* < 0.0001; unpaired Student’s *t* test. (M and N) Representative histogram overlay and MFI bar graphs of TNF-α, IFN-γ, and GRZMB in CD4^+^ and CD8^+^ T cells from spleens of *Dnmt1*^flox/flox^*Cd4*^cre/+^ and *Dnmt1*^flox/flox^ control mice at day 7 after cardiac transplantation. Numbers on the right indicate MFI values for respective colored samples. ∗∗∗*p* < 0.001 and ∗∗∗∗*p* < 0.0001; unpaired Student’s *t* test. All results are expressed as the mean ± standard deviation, with *n* = 5 unless otherwise specified.

To explore the impact of DNMT1 on T cells in heart transplantation, we collected splenocytes and graft-infiltrating cells for analysis on day 7 after transplantation in a mouse heart transplant model. The results showed that, on day 7 post-transplantation, T cells in the graft of *Dnmt1*^flox/flox^ *Cd4*^cre/+^ mice were significantly reduced compared with the control group (Figures 2A, 2B, and S5D), which might account for the reason for long-term survival in mouse heart transplantation. Graft infiltrating CD4^+^ T and CD8^+^ T cells’ effector functions (CD44^+^CD62L^−^) were also diminished in *Dnmt1*^flox/flox^ *Cd4*^cre/+^ mice (Figures 2C, 2D, and S5E). Naive TCF1^+^ T cells came to TCF1^−^CX3CR1^+^ effector T cells upon alloantigen stimulus, while *Dnmt1*^flox/flox^ *Cd4*^cre/+^ mice also had lower proportions of TCF1^−^CX3CR1^+^ CD4^+^ T and CD8^+^ T cells (Figures 2E and 2F). The above results indicate that knockout of DNMT1 leads to a reduction in the effector differentiation of graft-infiltrating T cells in the acute cardiac rejection model.

As for the peripheral immune organ spleen, the percentage of CD4^+^ and CD8^+^ T cells decreased in *Dnmt1*^flox/flox^ *Cd4*^cre/+^ mice (Figures 2G, 2H, and S5F). The proportions of CD44^+^CD62L^−^ and TCF1^−^CX3CR1^+^ effector T cells decreased simultaneously (Figures 2I–2L and S5G). We also examined the expression of effector cytokines such as TNF-α, IFN-γ, and granzyme B. *Dnmt1*^flox/flox^ *Cd4*^cre/+^ CD4^+^ and CD8^+^ T cells had lower expression of TNF-α, IFN-γ, and granzyme B, indicating that, under acute antigen stimulation, the cells’ inflammatory cytokine secretion and cytotoxic function were reduced compared with the control group (Figures 2M and 2N).

### The absence of DNMT1 in CD4^+^ rather than in CD8^+^ T cells plays a crucial role in the rejection reaction

**Figure 3:**
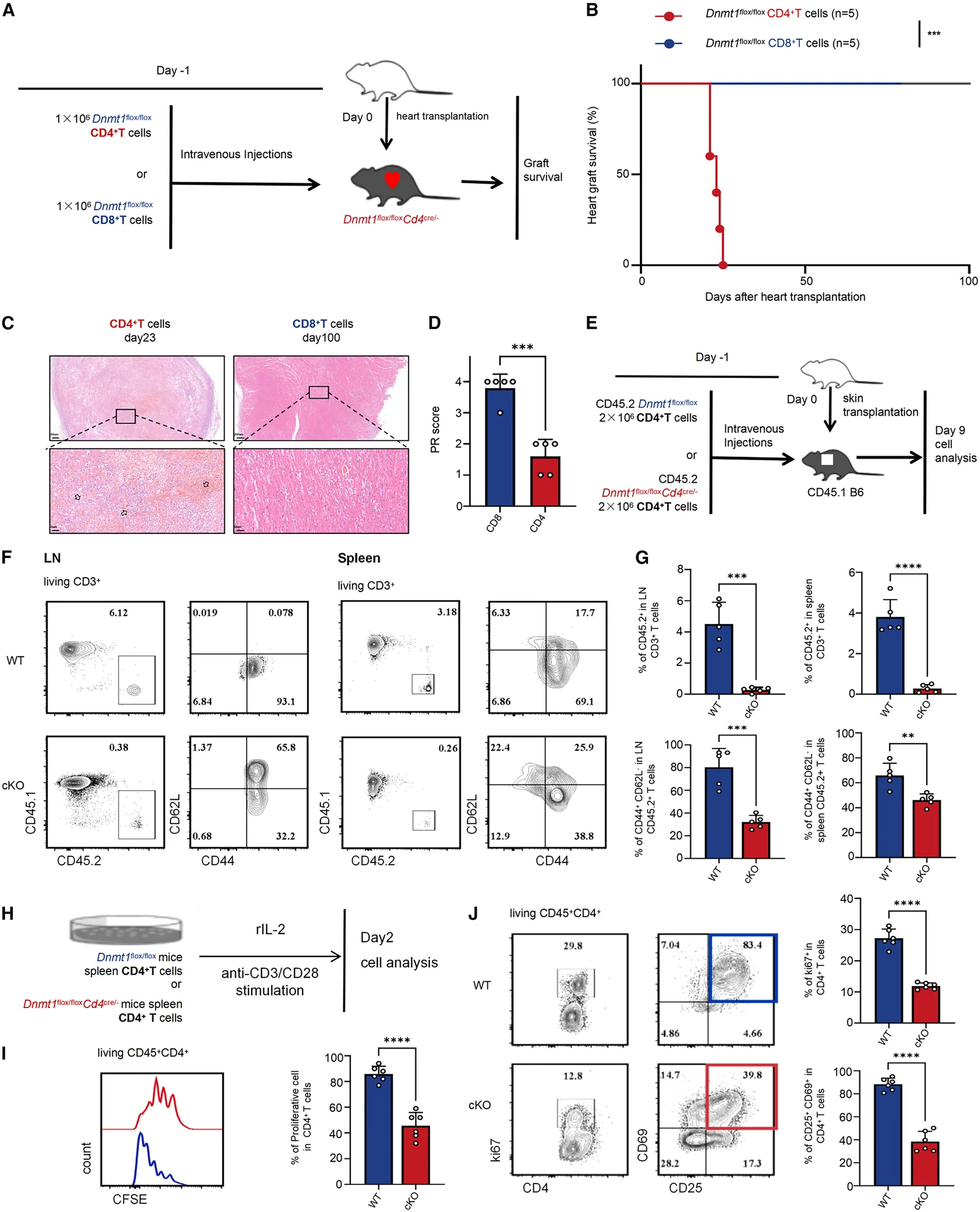
The absence of DNMT1 in CD4^+^ rather than CD8^+^ T cells plays a crucial role in the rejection reaction (A) CD4^+^ or CD8^+^ T cells were sorted from the spleens of *Dnmt1*^flox/flox^ mice and 5 × 10^6^ cells were injected intravenously into *Dnmt1*^flox/flox^*Cd4*^cre/+^ mice on day 0, followed by heart transplantation on day 1. (B) Percentage of heart and skin allograft survival of *Dnmt1*^flox/flox^*Cd4*^cre/+^ mice injected with CD4^+^ or CD8^+^ T cells from *Dnmt1*^flox/flox^ mice after transplantation. ∗∗∗*p* < 0.001; log rank test. (C and D) Representative images of H&E staining of grafts from *Dnmt1*^flox/flox^*Cd4*^cre/+^ mice into which *Dnmt1*^flox/flox^ CD4^+^ or CD8^+^ T cells were transferred after cardiac transplantation. Original magnification ×50 and ×200. Bar graphs of PR score at day 7 after cardiac transplantation. ∗∗∗*p* < 0.001; unpaired Student’s *t* test. (E) CD4^+^ cells were sorted from the spleens of *Dnmt1*^flox/flox^*Cd4*^cre/+^ and littermate control mice and transferred into CD45.1 congenic mice. The next day, we performed tail skin transplantation. On day 9 post-transplantation, we used flow cytometry to examine T cells in the spleen and draining lymph nodes. (F and G) Representative contour plots and bar graphs showing the percentage of transferred CD45.2^+^ T cells from *Dnmt1*^flox/flox^*Cd4*^cre/+^ and *Dnmt1*^flox/flox^ control mice in CD3^+^ cells and the percentage of CD44^+^CD62L^−^ cells in transferred CD45.2^+^ T cells in recipient spleens and draining lymph node at day 9 after skin transplantation. ∗∗*p* < 0.01, ∗∗∗*p* < 0.001, and ∗∗∗∗*p* < 0.0001; unpaired Student’s *t* test. (H) CD4^+^ T cells were sorted from spleen of *Dnmt1*^flox/flox^*Cd4*^cre/+^ and *Dnmt1*^flox/flox^ control mice and stimulated with anti-CD3/CD28 beads *in vitro* as well as the recombined IL-2. On day 2 after stimulation, we used flow cytometry to examine the T cells. (I) Representative histogram overlay of carboxyfluorescein succinimidyl ester (CFSE) and the bar graphs of percentage of proliferative cells in CD4^+^ T cells from *Dnmt1*^flox/flox^*Cd4*^cre/+^ and *Dnmt1*^flox/flox^ control mice after stimulation *in vitro*. *n* = 6, ∗∗∗∗*p* < 0.0001; unpaired Student’s *t* test. (J) Representative contour plots and bar graphs showing the percentage of Ki-67^+^ and CD25^+^CD69^+^ T cells in CD4^+^ T cells from *Dnmt1*^flox/flox^*Cd4*^cre/+^ and *Dnmt1*^flox/flox^ control mice at day 2 after stimulation *in vitro*. *n* = 6, ∗∗∗∗*p* < 0.0001; unpaired Student’s *t* test. All results are expressed as the mean ± standard deviation, with *n* = 5 unless otherwise specified.

To determine whether the absence of DNMT1 in CD4^+^ or CD8^+^ T cells plays a dominant role, we sorted CD4^+^ or CD8^+^ T cells from the spleens of *Dnmt1*^flox/flox^ mice and injected 5 × 10^6^ cells intravenously into *Dnmt1*^flox/flox^ *Cd4*^cre/+^ mice on day 0, followed by heart transplantation on day 1 (Figure 3A). The results showed that recipient mice infused with CD4^+^ T cells had a significantly reduced graft survival time, while those infused with CD8^+^ T cells showed no significant effect (Figure 3B). *Dnmt1*^flox/flox^ *Cd4*^cre/+^ mice that received *Dnmt1*^flox/flox^ CD4^+^ T cells had a greater number of inflammatory cells infiltrating the graft compared with those that received *Dnmt1*^flox/flox^ CD8^+^ T cells (Figure 3C). At the time of cardiac arrest or sacrifice, the grafts from mice that received *Dnmt1*^flox/flox^ CD4^+^ T cells had significantly lower PR scores than those that received CD8^+^ T cells (Figure 3D). This suggests that the absence of DNMT1 in CD4^+^, rather than CD8^+^, T cells plays a dominant role in heart transplant rejection.

To further verify the impact of DNMT1 knockout on CD4^+^ T cells, we isolated CD4^+^ cells from the spleens of *Dnmt1*^flox/flox^ *Cd4*^cre/+^ and littermate control mice and transferred them into CD45.1 congenic mice. The next day, we performed tail skin transplantation. On day 9 post-transplantation, we used flow cytometry to examine T cells in the spleen and draining lymph nodes (LNs) (Figure 3E). The results showed that T cells from *Dnmt1*^flox/flox^ *Cd4*^cre/+^ mice were significantly fewer in the spleen and lymph nodes compared with the control group, and the effector differentiation of *Dnmt1*^flox/flox^ *Cd4*^cre/+^ T cells was reduced (CD44^+^CD62L^−^) (Figures 3F and 3G).

Furthermore, our *in vitro* T cell activation assay revealed that, by the second day of anti-CD3/CD28 stimulation in the *in vitro* activation culture (Figure 3H), the proliferation ability of *Dnmt1*^flox/flox^ *Cd4*^cre/+^ CD4^+^ T cells was diminished. This was accompanied by a decrease in Ki-67 expression and a reduced proportion of effector subsets (CD25^+^CD69^+^) (Figures 3I and 3J). The absence of DNMT1 resulted in a notable decline in both the *in vivo* and the *in vitro* proliferation and activation capabilities of CD4^+^ T cells.

### T cell-specific knockout of DNMT1 can lead to T cell senescence in the cardiac transplantation

**Figure 4:**
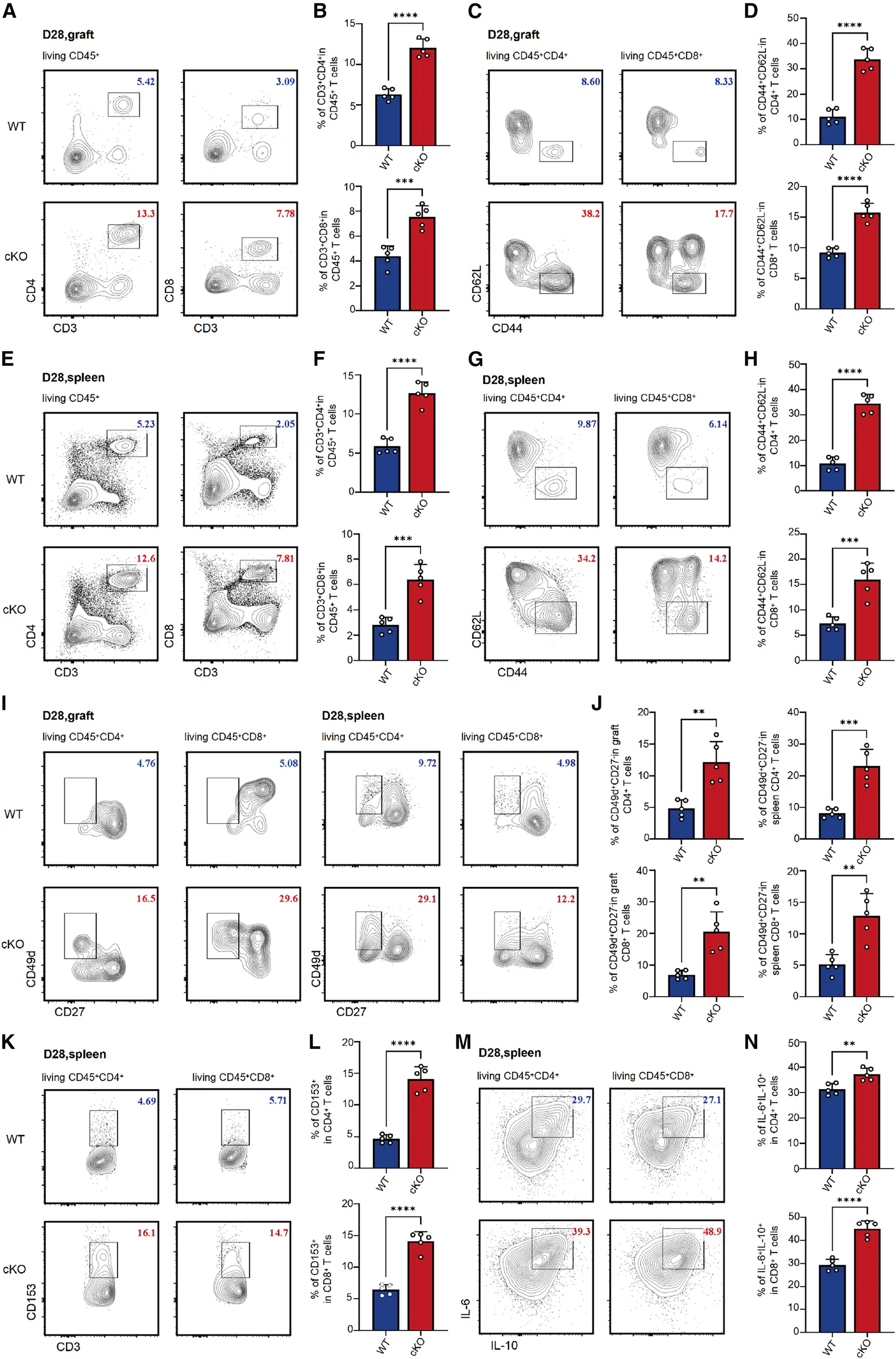
T cell-specific knockout of DNMT1 can lead to T cell senescence in the cardiac transplantation (A and B) Representative contour plots and bar graphs showing the percentage of CD4^+^ and CD8^+^ T cells in CD45^+^ cells of grafts from *Dnmt1*^flox/flox^*Cd4*^cre/+^ and *Dnmt1*^flox/flox^ control mice at day 28 after cardiac transplantation. ∗∗∗*p* < 0.001 and ∗∗∗∗*p* < 0.0001; unpaired Student’s *t* test. (C and D) Representative contour plots and bar graphs showing the percentage of CD44^+^CD62L^−^ T cells in CD4^+^ and CD8^+^ T cells of grafts from *Dnmt1*^flox/flox^*Cd4*^cre/+^ and *Dnmt1*^flox/flox^ control mice at day 28 after cardiac transplantation. ∗∗∗∗*p* < 0.0001; unpaired Student’s *t* test. (E and F) Representative contour plots and bar graphs showing the percentage of CD4^+^ and CD8^+^ T cells in CD45^+^ cells of spleens from *Dnmt1*^flox/flox^*Cd4*^cre/+^ and *Dnmt1*^flox/flox^ control mice at day 28 after cardiac transplantation. ∗∗∗*p* < 0.001 and ∗∗∗∗*p* < 0.0001; unpaired Student’s *t* test. (G and H) Representative contour plots and bar graphs showing the percentage of CD44^+^CD62L^−^ T cells in CD4^+^ and CD8^+^ T cells of spleens from *Dnmt1*^flox/flox^*Cd4*^cre/+^ and *Dnmt1*^flox/flox^ control mice at day 28 after cardiac transplantation. ∗∗∗*p* < 0.001 and ∗∗∗∗*p* < 0.0001; unpaired Student’s *t* test. (I and J) Representative contour plots and bar graphs showing the percentage of CD49d^+^CD27^−^ T cells in CD4^+^ and CD8^+^ T cells of spleens and grafts from *Dnmt1*^flox/flox^*Cd4*^cre/+^ and *Dnmt1*^flox/flox^ control mice at day 28 after cardiac transplantation. ∗∗*p* < 0.01 and ∗∗∗*p* < 0.001; unpaired Student’s *t* test. (K and L) Representative contour plots and bar graphs showing the percentage of CD153^+^ T cells in CD4^+^ and CD8^+^ T cells of spleens from *Dnmt1*^flox/flox^*Cd4*^cre/+^ and *Dnmt1*^flox/flox^ control mice at day 28 after cardiac transplantation. ∗∗∗∗*p* < 0.0001; unpaired Student’s *t* test. (M and N) Representative contour plots and bar graphs showing the percentage of IL-6^+^IL-10^+^ T cells in CD4^+^ and CD8^+^ T cells of spleens from *Dnmt1*^flox/flox^*Cd4*^cre/+^ and *Dnmt1*^flox/flox^ control mice at day 28 after cardiac transplantation. ∗∗*p* < 0.01 and ∗∗∗∗*p* < 0.0001; unpaired Student’s *t* test. All results are expressed as the mean ± standard deviation, with *n* = 5 unless otherwise specified.

To further investigate the impact of DNMT1-specific knockout on T cells in heart transplantation, on day 28 post-transplantation, splenocytes and graft-infiltrating cells were collected. Unlike on day 7, the proportion of T cells in *Dnmt1*^flox/flox^ *Cd4*^cre/+^ mice showed a reversal in both the spleen and the graft (Figures 4A, 4B, 4E, 4F, S5H, and S5J). This was mainly due to the fact that the grafts in the control group had been completely rejected and organized by day 28, while the *Dnmt1*^flox/flox^ *Cd4*^cre/+^ mice continued to receive antigenic stimulation. In the graft, the proportion of the TEM subset of T cells (CD44^+^CD62L^−^) in *Dnmt1*^flox/flox^ *Cd4*^cre/+^ mice was higher than that in the control group (Figures 4C, 4D, 4G, and 4H). Interestingly, the proportion of senescent T cells (CD49d^+^CD27^−^; CD49d^+^CD153^+^) in *Dnmt1*^flox/flox^ *Cd4*^cre/+^ mouse grafts was significantly increased compared with the control group (Figures 4I and S5I). In the spleen, the findings were consistent with those observed in the grafts. *Dnmt1*^flox/flox^ *Cd4*^cre/+^ mice had a higher proportion of CD49d^+^CD27^−^ and CD153^+^ senescent T cells compared with controls (Figures 4I–4L and S5K), which suggested that *Dnmt1*^flox/flox^ *Cd4*^cre/+^ T cells may undergo cellular senescence more rapidly after heart transplantation. Additionally, the expression of cytokines such as IL-6, IL-10, TNF-α, and IFN-γ was elevated, which is characteristic of the senescence-associated secretory phenotype (SASP) (Figures 4M, 4N, and S5). Furthermore, the upregulation of inhibitory receptors PD-1 and Tim3 on T cells from *Dnmt1*^flox/flox^ *Cd4*^cre/+^ mice, along with an increased activity of senescence-associated β-galactosidase, further supports the hypothesis of accelerated T cell senescence in these mice (Figure S5). These results collectively indicate that *Dnmt1*^flox/flox^ *Cd4*^cre/+^ T cells have undergone senescence in the heart transplant model.

### T cell-specific knockout of DNMT1 can lead to decreased methylation levels of the CDKN1A promoter and upregulate CDKN1A expression

**Figure 5:**
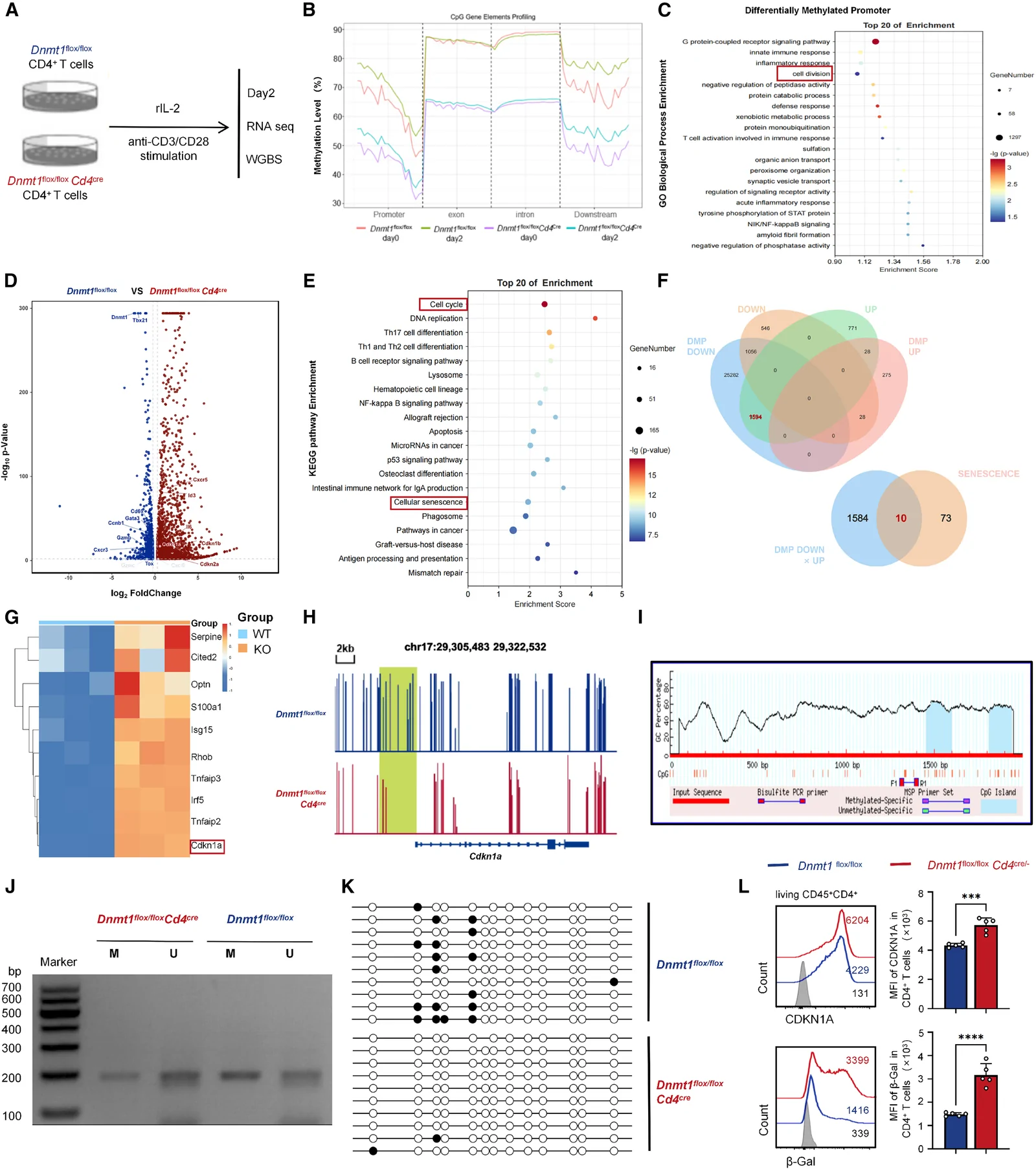
T cell-specific knockout of DNMT1 can lead to decreased methylation levels of the CDKN1A promoter and upregulate CDKN1A expression (A) *Dnmt1*^flox/flox^*Cd4*^cre/+^ and *Dnmt1*^flox/flox^ CD4^+^ T cells were collected on the second day of *in vitro* activation, and whole-genome bisulfite sequencing (WGBS) and RNA-seq analysis were performed. (B) Profiling map of percentage methylation level of CpG gene elements. (C) Bubble chart of pathways enriched in genes associated with differentially methylated promoters. (D and E) Volcano plot of differentially expressed genes in *Dnmt1*^flox/flox^*Cd4*^cre/+^ and *Dnmt1*^flox/flox^ CD4^+^ T cells on day 2 after *in vitro* stimulation and bubble chart of pathways enriched in these genes. (F) Venn diagram showing the overlap between upregulated and downregulated genes and genes with increased and decreased promoter methylation levels. (G) Heatmap of 871 genes with decreased promoter methylation levels and upregulated gene expression. (H) IGV visualization peak plot of *Cdkn1a* and its promoter methylation levels. (I) Schematic of CpG island prediction of the CDKN1A promoter and methylation primer design. (J) Image of electrophoresis result of methylation-specific PCR of *Dnmt1*^flox/flox^*Cd4*^cre/+^ and *Dnmt1*^flox/flox^ CD4^+^ T cells on day 2 after *in vitro* stimulation. (K) Representative dot plot of CDKN1A promoter methylation levels obtained by bisulfite sequencing PCR (BSP). Each row of dots represents one sequenced clone, and each dot represents one CpG site. Ten clones were sequenced per sample. Empty or dark dots indicate unmethylated or methylated CpGs. All BSP experiments were performed in three independent biological replicates. (L) Representative histogram overlay and MFI bar graphs of CDKN1A and β-Gal in CD4^+^ T cells from *Dnmt1*^flox/flox^*Cd4*^cre/+^ and *Dnmt1*^flox/flox^ control mice at day 2 after stimulation *in vitro*. Results are expressed as the mean ± standard deviation. Numbers on the right indicate MFI values for respective colored samples. *n* = 5, ∗∗∗*p* < 0.001 and ∗∗∗∗*p* < 0.0001; unpaired Student’s *t* test.

To explore the regulatory mechanisms of DNMT1-specific knockout on the functional changes in CD4^+^ T cells, we collected *Dnmt1*^flox/flox^ *Cd4*^cre/+^ and *Dnmt1*^flox/flox^ CD4^+^ T cells on the second day of *in vitro* activation and performed whole-genome bisulfite sequencing (WGBS) and RNA-seq analysis (Figure 5A). The results showed that, due to the absence of DNMT1, the average methylation levels of various genomic elements in *Dnmt1*^flox/flox^ *Cd4*^cre/+^ CD4^+^ T cells were reduced (Figure 5B). Differential methylation promoters were enriched in cell division pathways (Figure 5C). The differentially expressed genes were enriched in negative regulation of cell-cycle pathways, and the expression of cell-cycle inhibitory molecules, including CDKN1A, was increased (Figures 5D and 5E). These indicated that, on the second day of *in vitro* activation, *Dnmt1*^flox/flox^ CD4^+^ T cells exhibited cell-cycle arrest, which is a significant characteristic of cellular senescence.

To identify differentially expressed genes due to differential methylation promoters, we found 1,594 genes in common in WGBS and RNA-seq. Among these genes, there were 10 that were related to cellular senescence (Figures 5F and 5G). CDKN1A, as a direct regulator of senescence-associated cell-cycle arrest, has more direct evidence for regulating T cell senescence among the these 10 genes.^12^ To more intuitively demonstrate the differences in the methylation levels of the CDKN1A promoter, Integrative Genomics Viewer (IGV) was used to visualize the results of WGBS, and we found that the methylation peaks on the CDKN1A promoter were reduced (Figure 5H). To further verify, MethPrimer was used to predict the location of CpG islands and to design methylation primers (Figure 5I). Methylation-specific PCR and bisulfite sequencing PCR were used to validate the sequencing results, and it was observed that the methylation level of the CDKN1A promoter in *Dnmt1*^flox/flox^ *Cd4*^cre/+^ CD4^+^ T cells was reduced (Figures 5J and 5K). Flow cytometry results revealed that, on day 2 of *in vitro* stimulation, the expression of CDKN1A and the activity of senescence-associated β-galactosidase in T cells from *Dnmt1*^flox/flox^ *Cd4*^cre/+^ mice were upregulated (Figure 5L). These results were also observed in spleens and grafts on day 28 after heart transplantation (Figure S6). Furthermore, when DNMT1 expression in 293T cells was silenced using lentivirus, a similar reduction in CDKN1A promoter methylation was observed, proving that DNMT1 can also regulate the methylation level of the CDKN1A promoter in human cells (Figure S7). These results suggest that DNMT1 ablation may lead to T cell senescence through the CDKN1A pathway.

### Depletion of CDKN1A^high^ cells can reverse T cell senescence induced by 5-Azac

**Figure 6:**
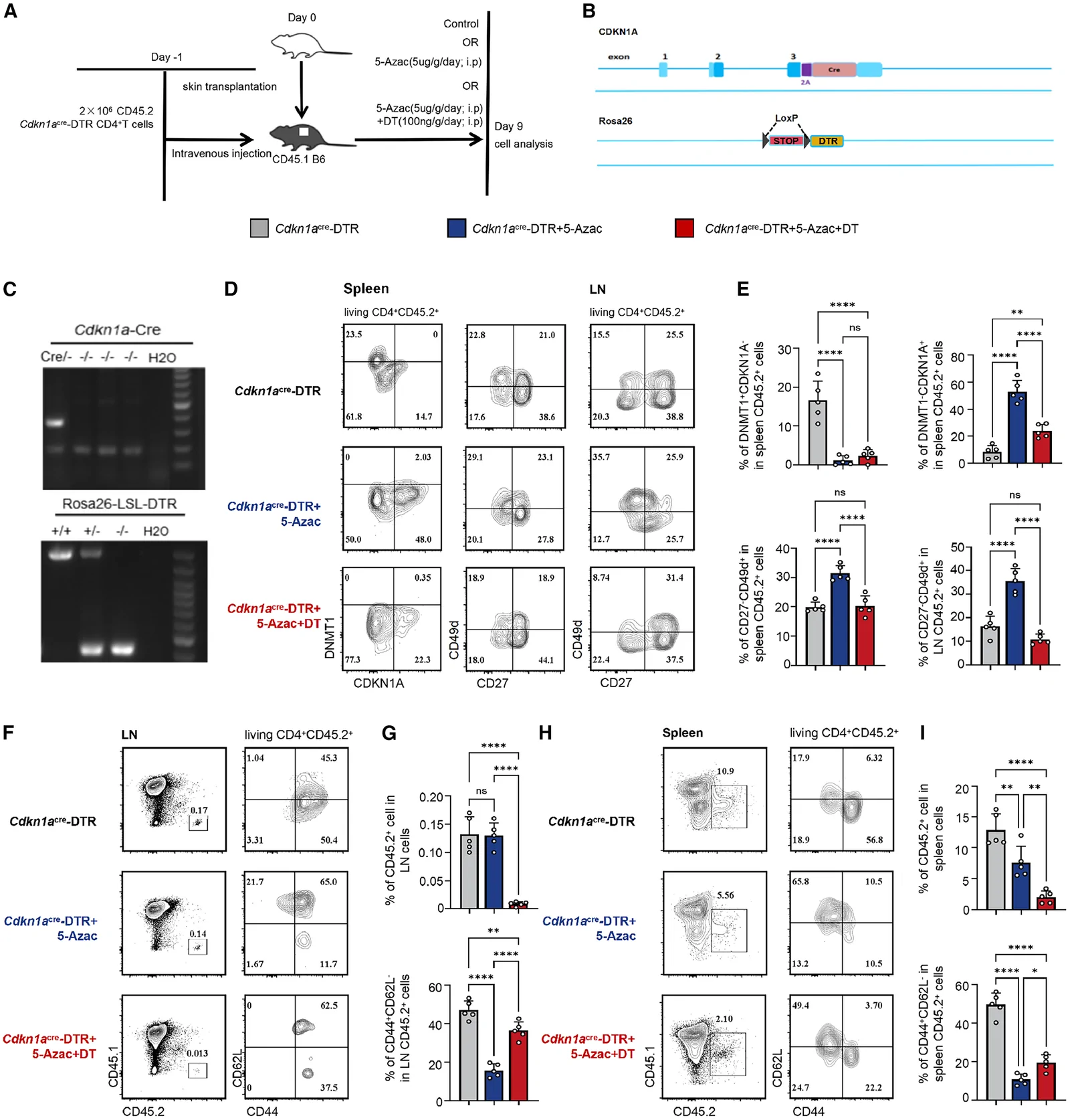
Depletion of CDKN1A^high^ cells can reverse T cell senescence induced by 5-azacytidine (A) CD45.2 *Cdkn1a*^cre^-DTR CD4^+^ T cells (2 × 10^6^) were transferred into CD45.1 mice, followed by skin transplantation. Saline solution, 5-Azac, or 5-Azac + DT was injected into the CD45.1 recipient mice. Flow cytometry was used to analyze mouse spleens and draining lymph nodes on day 9. (B) Gene schematic for *Cdkn1a*^cre^-DTR mice. A flox STOP cassette was inserted in front of the diphtheria toxin receptor sequence, which can be removed by Cre recombinase driven by the CDKN1A promoter. (C) Representative agarose gel electrophoresis image for *Cdkn1a*^cre^-DTR mouse tail gene identification. (D and E) Representative contour plots and bar graphs showing the percentage of CDKN1A^+^DNMT1^−^, CDKN1A^−^DNMT1^+^, and CD49d^+^CD27^−^ cells in CD4^+^ and CD8^+^ T cells of spleens and draining lymph nodes. ns, no significance; ∗∗*p* < 0.01 and ∗∗∗∗*p* < 0.0001; multiple comparisons following ANOVA. (F and G) Representative contour plots and bar graphs showing the percentage of transferred CD45.2^+^ T cells in CD3^+^ cells and the percentage of CD44^+^CD62L^−^ cells in transferred CD45.2^+^ T cells in recipient draining lymph nodes at day 9 after skin transplantation. ns, no significance; ∗∗*p* < 0.01 and ∗∗∗∗*p* < 0.0001; multiple comparisons following ANOVA. (H and I) Representative contour plots and bar graphs showing the percentage of transferred CD45.2^+^ T cells in CD3^+^ cells and the percentage of CD44^+^CD62L^−^ cells in transferred CD45.2^+^ T cells in recipient spleens at day 9 after skin transplantation. ∗*p* < 0.05, ∗∗*p* < 0.01, and ∗∗∗∗*p* < 0.0001; multiple comparisons following ANOVA. All results are expressed as the mean ± standard deviation, with *n* = 5 unless otherwise specified.

To further validate the regulation mechanism of CDKN1A by DNMT1 *in vivo*, we constructed *Cdkn1a*^cre^-DTR mice, in which a flox STOP cassette was inserted in front of the diphtheria toxin receptor (DTR) sequence, which can be removed by Cre recombinase driven by the CDKN1A promoter. Consequently, CDKN1A^high^ cells can express the DTR, and these cells can be eliminated by the administration of diphtheria toxin (Figures 6A–6C). 5-Azac is a potent DNMT1 inhibitor that can suppress the expression and activity of DNMT1. Due to the need for using diphtheria toxin and considering the stability of the model, we transferred CD4^+^ T cells from CD45.2 *Cdkn1a*^cre^-DTR mice into CD45.1 congenic mice and performed skin transplantation to assess the changes in T cells *in vivo*. Mice were randomly divided into three groups with or without the injection with 5-Azac and diphtheria toxin (DT). On day 9 post-transplantation, we examined the status of *Cdkn1a*^cre^-DTR cells in the lymph nodes and spleen. The results showed that injection of 5-Azac can induce high expression of CDKN1A in CD4^+^ T cells, while injection of diphtheria toxin can eliminate CDKN1A^high^ cells, and diphtheria toxin could reverse the cellular senescence (CD27^−^CD49d^+^) caused by 5-Azac in the spleen and lymph nodes (Figures 6D and 6E). Otherwise, despite the significant reduction in the number of transferred cells in the spleen and lymph nodes after the depletion of CDKN1A^high^ cells, the proportion of effector cells (CD44^+^CD62L^−^) increased (Figures 6F–6I). This indicated that depletion of CDKN1A^high^ cells can reverse T cell senescence induced by DNMT1 inhibition.

### Heart transplant recipients with long-term tolerance exhibit increased levels of T cell senescence

**Figure 7:**
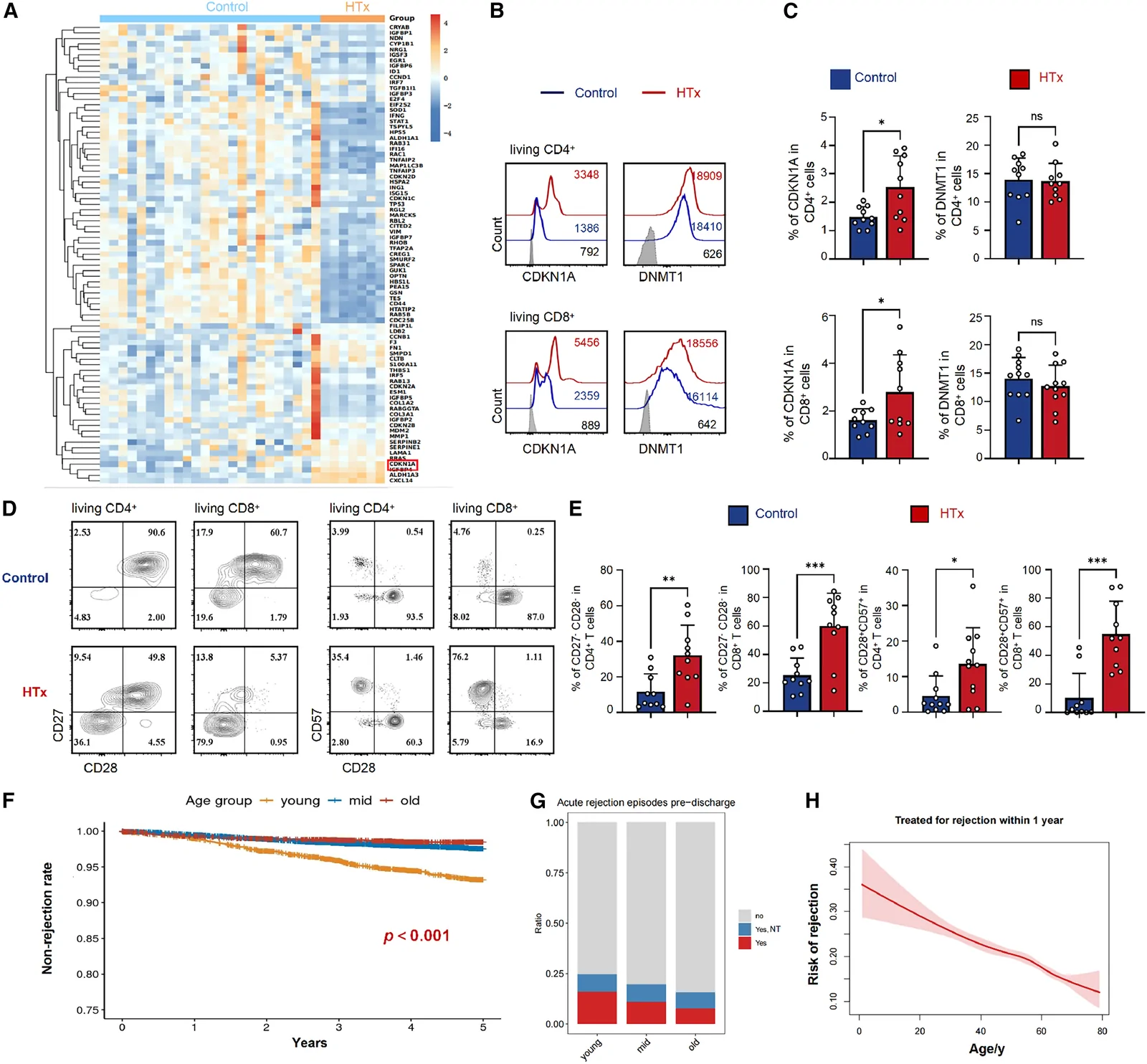
Heart transplant recipients with long-term tolerance exhibit increased levels of T cell senescence (A) Heatmap of the genes chosen by senescence-associated gene sets in the MSig database of peripheral blood from tolerant patients and healthy controls of similar age in the GEO database’s GSE5967 and GSE6613. (B and C) Representative histogram overlay and MFI bar graphs of CDKN1A and DNMT1 in CD4^+^ and CD8^+^ T cells from patients with heart transplantation and healthy controls. Numbers on the right indicate MFI values for respective colored samples. ns, no significance; ∗*p* < 0.05; unpaired Student’s *t* test. (D and E) Representative contour plots and bar graphs showing the percentage of CD27^−^CD28^−^ and CD28^−^CD57^+^ cells in CD4^+^ and CD8^+^ T cells from patients with heart transplantation and healthy controls. ∗*p* < 0.05, ∗∗*p* < 0.01, and ∗∗∗*p* < 0.001; unpaired Student’s *t* test. All results are expressed as the mean ± standard deviation, with *n* = 10. (F) Kaplan-Meier curve of rejection-free rate over time stratified by age groups of patients in the UNOS database. Groups: young (18–40 years old), middle-aged (40–65 years old), and elderly (>65 years old). Log rank test. (G) Stacked bar chart of the incidence of acute rejection reactions within 1 year of heart transplantation of patients in the UNOS database. No, no rejection; Yes-NT, rejection with no treatment; Yes, rejection with treatment. (H) Logistic regression curve for age and the likelihood of requiring treatment for rejection within 1 year of transplantation.

We then used clinical cohorts and an online database to verify the relationship between T cell senescence and human heart transplantation. By identifying senescence-associated gene sets in the MSig database and comparing the gene expression profiles of peripheral blood from tolerant patients and healthy controls of similar age in the GEO database’s GSE5967 and GSE6613, we found that the level of CDKN1A in peripheral blood cells of tolerant patients is increased when CD3E is used as the reference gene (Figure 7A). In the clinical cohorts of Wuhan Union Hospital, we examined peripheral blood mononuclear cells (PBMCs) from heart transplant recipients who had survived more than 1 year without rejection and controls using flow cytometry. The baseline characteristics are listed in Table S2. Though the change in DNMT1 was not significant between the groups, the expression of CDKN1A increased significantly (Figures 7B and 7C), and the level of senescence in CD4^+^ and CD8^+^ T cells (CD28^+^CD57^+^, CD28^−^CD27^−^) of patients without rejection was also increased (Figures 7D and 7E).

There is a highly significant positive correlation between age and T cell senescence. As age increases, T cells undergo a series of age-related changes, such as increased DNA damage, short telomeres, loss of CD28, and increased production of inflammatory cytokines.^13^^,^^14^ To further validate the relationship between T cell senescence and human heart transplant rejection, United Network for Organ Sharing (UNOS) data for all patients who underwent heart transplantation from January 2005 to December 2021 were also included. A total of 20,773 adult heart transplant recipients were stratified into three distinct age groups: young (18–40 years old), middle-aged (40–65 years old), and elderly (>65 years old). The baseline characteristics of donors and recipients are listed in Table S3. Survival analysis showed that, as age increases, the incidence of rejection reactions decreases (Figures 7F and 7G). To adjust for potential confounders, a multivariable Cox regression analysis identified several factors independently associated with graft failure due to rejection (Table S4). Recipient age was a strong predictor, with young recipients having a significantly higher risk (hazard ratio [HR] 2.69, 95% confidence interval [CI] 2.20–3.29, *p* < 0.001) and older recipients having a lower risk (HR 0.67, 95% CI 0.49–0.91, *p* = 0.010) compared with the middle-aged reference group. The composite donor risk index, total bilirubin, creatinine, dialysis at listing, pre-operative extracorporeal membrane oxygenation (ECMO), and pre-operative ventilator support were not significantly associated with the risk of graft failure due to rejection in this model. Furthermore, to explore the relationship between age and risk of rejection, a logistic regression analysis was conducted. There is a significant association between age group and the likelihood of requiring treatment for rejection within 1 year of transplantation (Figure 7H). The above results suggest that T cell senescence plays a role in long-term tolerance in heart transplant patients.

## Discussion

DNMT1 primarily functions to maintain methylation, that is, to preserve the methylation status at the same positions on the daughter strand as on the parent strand during semi-conservative DNA replication.^15^ Under normal circumstances, T cells are rapidly activated upon stimulation by allogeneic antigens and mediate graft rejection reactions.^2^ We found that, due to the absence of DNMT1, the high methylation level of the CDKN1A promoter was not maintained during semi-conservative replication. The epigenetic silencing of CDKN1A was relieved, leading to the accumulation of CDKN1A in T cells, which in turn leads to cellular senescence and mitigates graft rejection reactions. This was manifested as long-term graft survival (>100 days) in the mouse cardiac transplantation model. Clinical data from UNOS showed that elderly transplant patients have a lower incidence of rejection reactions, with T cell senescence playing a role in this process.

Total knockout of DNMT1 leads to embryonic death in mice.^16^ Knocking out DNMT1 in adult mice also results in rapid death, and the dying mice exhibit severe hematopoietic dysfunction.^17^ Unlike the stable proliferative capacity of control cells, a decreasing proliferative capacity was observed in consecutive co-transfer experiments by reducing DNMT1 levels through hypomorphic mutations.^17^ Lymphocyte protein tyrosine kinase (Lck) controls T cell proliferation and antigen-related activation responses in the early stages of T cell thymic development.^18^ By using the Lck promoter to drive Cre recombinase, knocking out DNMT1 in the early stages of thymic development leads to a significant loss of double-positive and single-positive cells in the thymus, resulting in even fewer cells reaching the periphery. However, using the CD4 promoter to drive Cre recombinase and knocking out DNMT1 in T cells after the rapid thymic proliferation stage does not significantly affect the number of cells reaching the periphery.^6^ Interestingly, the proliferative capacity of the peripheral T cells decreased, while their capacity to secrete increased, which is consistent with the phenomenon of cellular senescence and the SASP that we observed in *Dnmt1*^flox/flox^ *Cd4*^cre/+^ T cells in the mouse cardiac transplantation model.

Under internal and external stimulation or normal physiological states, almost all somatic cells undergo senescence, which is manifested as long-term irreversible cycle arrest, SASP, damage to macromolecules, metabolic abnormalities, and so on.^19^ As a type of somatic cell, T cells also possess all the characteristics of somatic cell senescence, which endows them with poor antigen clearance capabilities.^20^ In this study, *Dnmt1*^flox/flox^ *Cd4*^cre/+^ T cells showed significant proliferation inhibition, and the activity of senescence-associated β-galactosidase was also significantly increased compared with the control group.

Senescent T cells characteristically express surface markers. For example, both humans and mice show increased expression of NK cell-related markers (KLRG1 and NKG2A).^21^ In humans, senescent T cells lack the expression of co-stimulatory receptors CD27 and CD28 and show high expression of CD57.^22^ At the same time, they lose lymphoid homing receptors CD62L and CCR7, making the cells more inclined to enrich in the periphery.^23^ The immunosenescence markers of human T cells, such as the absence of CD28 and CD57 and the re-expression of CD45RA, are not applicable to mice, making it more difficult to mark the characteristics of senescent cells in mice.^24^ The characterization of senescent T cells in mice tends to choose the shift in the senescence-associated cellular differentiation pool; that is, the proportion of terminal TEM (CD44^+^CD62L^−^) T cell subsets increases with age.^25^^,^^26^ In fact, in studies on humans, there are also cases of using terminal TEM cells to mark senescent T cells.^27^ However, in acute responses, this kind of marking is obviously not appropriate. In addition, the decrease in CD27 expression and the accumulation of KLRG1^+^ cells in aging mice are similar to those in humans.^28^ Through single-cell sequencing, some studies have found that the expression of CD49d is related to senescence-associated CD8^+^ T cells in tissues and can be used as a characteristic marker of senescence-associated T cells.^29^ In this study, we observed that *Dnmt1*^flox/flox^ *Cd4*^cre/+^ T cells exhibited a molecular expression pattern of senescence, including the downregulation of CD27, the upregulation of CD49d, and an increase in secretory factors such as IL-6, IL-10, TNF-α, and IFN-γ. CD153 is a classic marker of senescent T cells, and a vaccine targeting CD153 has been developed to combat T cell senescence.^26^^,^^30^ At the same time, the expression of surface co-inhibitory receptors on senescent T cells will be upregulated, and some co-inhibitory molecules, such as PD1, TIM3, and TIGIT, have been confirmed to be involved in the process of T cell senescence.^31^^,^^32^^,^^33^ These were also observed in our study.

CDKN1A plays a crucial role in cellular senescence. By inhibiting various CDKs (cyclin-dependent kinases), such as CDK2, CDK4, and CDK6, the accumulation of CDKN1A can halt the progression of the cell cycle, leading to cell-cycle arrest in the G1 phase or G2/M phase, while inhibiting the degradation of CDKN1A can induce cellular senescence.^34^ Additionally, CDKN1A can regulate the expression of SASP, which is referred to as p21-activated secretory phenotype (PASP), to induce the clearance of senescent cells by the immune system.^35^ The clearance of cells with high CDKN1A expression has been proven to extend the lifespan of individuals and improve the function of aged organs.^36^ Moreover, CDKN1A can combine with the transcriptional repressor Zbtb18 to synergistically inhibit the expression of cKit, thereby regulating the self-renewal capacity of hematopoietic stem cells.^37^ In studies targeting bladder cancer, the methylation level of the CDKN1A promoter in human bladder cancer cell lines T24 and J82 can be observed to change by introducing an overexpression plasmid of DNMT1. By knocking down or overexpressing DNMT1, the protein level of CDKN1A in cells shows an inverse trend compared with that of DNMT1.^38^ Similarly, inhibiting the expression of DNMT1 in squamous carcinoma cells can lead to an increase in CDKN1A expression.^39^ The use of the DNMT inhibitor 5-Azac can successfully induce senescence in fibroblasts.^40^ By specifically knocking out DNMT1 in T cells, we also found an upregulation of CDKN1A expression in a cardiac transplantation model, leading to T cell senescence. Clearing cells with high CDKN1A expression can reverse the T cell phenotype induced by the DNMT1 inhibitor 5-Azac. These results indicate the presence of a DNMT1-CDKN1A-cellular senescence pathway in T cells, which could provide new therapeutic targets for clinical drug design.

In the present study, only Cdkn1a was functionally validated as a downstream target driving T cell senescence. The other nine genes that also emerged from our differential expression screen—Serpine1, Cited2, Optn, S100a1, Isg15, Rhob, Tnfaip3, Irf5, and Tnfaip2—were not individually manipulated or phenotyped. Consequently, two specific limitations arise: first, this may lead to functional redundancy or the overlooking of synergistic effects. Senescence is governed by parallel pathways. Tnfaip3 (A20) restrains NF-κB-dependent SASP, whereas Irf5 amplifies type-I-IFN-driven senescence programs; either could potentiate or counteract Cdkn1a-induced cell-cycle arrest. Without gain- or loss-of-function tests, we cannot determine whether these genes act redundantly or synergistically with CDKN1A.^41^ Second, it may lead to limited generalizability. Our conclusions derive from short-term CD3/CD28 + IL-2 stimulation. The consistency of this with the cellular senescence processes observed during natural aging *in vivo* and chronic antigenic stimulation in grafts remains to be evaluated, which limits the generalizability of our findings.

In summary, we demonstrated that, in heart transplantation, the absence of DNMT1 can lead to impaired T cell effector differentiation and accelerated senescence. Senescent T cells have a decreased ability to clear allogeneic antigens, which in turn leads to long-term survival of the heart graft (>100 days). This functional change in T cells is mediated by the loss of methylation levels at the CDKN1A promoter and the accumulation of CDKN1A. In conclusion, DNMT1 plays an important role in T cell-mediated alloreactive responses, and its association with T cell senescence is of significant importance in inducing transplant tolerance. Our data identify DNMT1 inhibition as a pharmacologically tractable strategy to induce CD4^+^ T cell senescence and thereby curtail cardiac allograft rejection. A short, low-dose course of an FDA-approved DNMT1 inhibitor (e.g., 5-Azac) could be readily translated to an early-phase trial in high-risk heart transplant recipients, with CDKN1A upregulation and senescent T cell abundance as companion biomarkers. Because CD4^+^ targeting is central, this epigenetic approach may offer a safer alternative to global T cell depletion or calcineurin inhibition.

## Materials and methods

### Mice

C57BL/6, B6.SJL CD45.1, and BALB/c mice were purchased from Shulaibao (Wuhan) Biotechnology Co., and *Dnmt1*^flox/flox^, Cd4^*cre*^, *Cdkn1a*^flox/flox^, and Rosa26-LSL-DTR mice (with the *SA-LSL-DTR-IRES* cassette inserted into the mouse Gt(ROSA)26Sor locus) were purchased from the Shanghai Model Organisms Center. *Cd4*^*cre*^ mice were bred with *Dnmt1*^flox/flox^ mice to generate *Dnmt1*^flox/flox^ *Cd4*^cre/+^ mice, and Rosa26-LSL-DTR mice were bred with *Cdkn1a*^flox/flox^ mice to generate *Cdkn1a*^flox/flox^-DTR mice and confirmed by PCR-assisted genotyping. Mice at 8 to 10 weeks of age were used for all studies as well as for heart transplantation.

All animal procedures complied with the “Guide for the Care and Use of Laboratory Animals” published by the US National Institutes of Health (NIH Publication No. 85-23, revised 1996) and were approved by the Animal Care and Use Committee of Tongji Medical College (IACUC NO: 3764). All animals were maintained under specific-pathogen-free conditions at Tongji Medical College, Huazhong University of Science and Technology, Wuhan, China.

### Skin and heart transplantation

Skin transplantation was performed as previously described.^42^ Briefly, full-thickness tail skin from donor mice (∼1 × 1 cm) was transplanted onto the dorsal flank of recipient mice and secured with a bandage. Rejection was defined as >90% of necrosis of the donor skin graft. The heart allografts from donor mice were transplanted into 8- to 10-week-old recipient mice as previously reported.^43^ Briefly, heart grafts were harvested from sex-matched donor mice and transplanted into the abdominal cavity of recipient mice by end-to-side anastomosing the aorta and pulmonary artery of the graft to the recipient’s aorta and vena cava, respectively. Graft survival was monitored by daily transabdominal palpation, and rejection was defined as a complete cessation of heartbeat.

### Tissue histology

Mice bearing a heart allograft were euthanized on various days post-transplantation, and grafts were removed and formalin-fixed for 48 h. Hematoxylin and eosin (H&E) staining was performed on paraffin sections of heart allografts. PR score was graded as described previously with the following scale: 0R (no rejection), 1R (focal mononuclear cell infiltrates without necrosis), 2R (focal mononuclear cell infiltrates with necrosis), 3R (multifocal infiltrates with necrosis), and 4R (widespread infiltrates with hemorrhage and vasculitis).

### Isolation of lymphoid cells from the mouse tissue and flow cytometric analysis

Mouse spleens and lymph nodes were directly isolated and then ground on a 40 μm filter mesh using the plunger of a 1 mL syringe. Red blood cell lysis buffer was used to remove red blood cells from the filtrate. For heart grafts, the digestion and separation methods were as previously described, followed by passing through a 40 μm strainer. The filtrate was slowly layered onto the upper layer of 44% Percoll and then centrifuged at 500*g*. The pellet was collected and prepared for various analyses. Fluorochrome-conjugated antibodies specific for mouse CD3 (17A2), CD4 (GK1.5), CD8 (53-6.7), CD62L (MEL-14), CD44 (IM7), CD25 (PC61.5), CD69 (H1.2F3), CD45 (30-F11), CD45.1 (A20), CD45.2 (104), KLRG1 (2F1/KLRG1), PD-1 (29F.1A12), TNF-α (MP6-XT22), perforin (S16009B), granzyme B (QA16A02), IFN-γ (XMG1.2), IL-6 (MP5-20F3), IL-10 (JES5-16E3), Ki-67 (16A8), CD153 (RM153), CD27 (LG.3A10), CD49d (R1-2), and CX3CR1 (SA011F11) were purchased from BioLegend, and antibody specific for CDKN1A (sc-6246) was purchased from Santa Cruz Biotechnology. Antibodies specific for human CD3 (SK7), CD45 (HI30), CD4 (RPA-T4), CD8 (HIT8a), CD27 (M-T271), CD28 (CD28.2), and CD57 (HNK-1) were purchased from BioLegend, and antibody specific for CDKN1A (ab282187) was purchased from Abcam. Fluorochrome-conjugated antibodies for TCF1 (C63D9) and DNMT1(64503) were purchased from Cell Signaling Technology.

For all staining, dead cells were excluded by using Zombie Aqua dye (BioLegend). Expression of transcription factor was stained for with a Foxp3 staining buffer set (00-5523-00; eBioscience), followed by staining with fluorochrome-conjugated antibodies. For intracellular cytokine staining, cells were briefly re-stimulated for 4 h with Cell Activation Cocktail (with Brefeldin A) (BioLegend) and then fixed and permeabilized with the Foxp3 staining buffer set and stained with fluorochrome-conjugated antibodies according to the manufacturers’ instructions. All samples were acquired with an LSR II flow cytometer (BD Biosciences), and the resulting data were processed using FlowJo software (Tree Star, Inc.).

A CFSE Cell Division Tracker Kit (BioLegend) was purchased to monitor cell proliferation according to the manufacturer’s instructions. The CellEvent Senescence Green Flow Cytometry Assay Kit (Thermo Fisher Scientific) was purchased to observe cell senescence according to the manufacturer’s instructions.

Cells were gated on FSC-A/SSC-A for viable lymphocytes, followed by FSC-A/FSC-H for doublet exclusion, and then on live cells using Zombie Aqua viability dye (Figure S4).

### Adoptive transfer of CD4^+^ T and CD8^+^ T cells

The Dynabeads Untouched Mouse CD4^+^ T Cells Kit (Thermo Fisher Scientific) was used to isolate specific CD4^+^ T cells from the spleen of CD45.2 littermate control *Dnmt1*^flox/flox^, CD45.2 *Dnmt1*^flox/flox^ *Cd4*^cre/+^ mice or *Cdkn1a*^flox/flox^-DTR mice. CD45.1 congenic mice were co-injected intravenously with a mixture of 5 × 10^6^ CD45.2 *Dnmt1*^flox/flox^, CD45.2 *Dnmt1*^flox/flox^ *Cd4*^cre/+^, or *Cdkn1a*^flox/flox^-DTR cells on day −1 and transplanted with BALB/c skin allografts on day 0. Next, flow cytometric analysis was performed on splenic or lymph node T cells on separate post-operative days.

Similarly, the Dynabeads Untouched Mouse CD8^+^ T Cells Kit (Thermo Fisher Scientific) was used to isolate 5 × 10^6^ CD8^+^ T cells from the spleen of *Dnmt1*^flox/flox^ mice. These isolated cells, along with the 5 × 10^6^ isolated *Dnmt1*^flox/flox^ CD4^+^ T cells, were injected via the tail vein into different *Dnmt1*^flox/flox^ *Cd4*^cre/+^ mice, and the mice were transplanted with a BALB/c heart on day 0 to observe the survival time of the graft.

### RNA-seq analysis

CD4^+^ T cells were sorted from splenocytes of wild-type (WT), *Dnmt1*^flox/flox^, or *Dnmt1*^flox/flox^ *Cd4*^cre/+^ mice, with or without *in vitro* stimulation, and stored in TRIzol (Thermo Fisher Scientific, Waltham, MA). Samples were then sent to Shanghai OE Biotech Co. for low-input total RNA library construction using SMART seq v.4 kit lib (Takara Inc., Mountain View, CA). The genes with RNA integrity number (≥8.0) were further sequenced on the Illumina HiSeq platform. Paired-end libraries with an average insert size of 150 bp were obtained. The RNA sequence qualities, including GC content and ErrorRate, were evaluated by the in-house pipeline from Novogene. Raw reads were aligned to a mouse reference (mm10) using STAR aligner (version 2.6). Fragments per kilobase of transcript per million mapped reads (FPKM) was calculated to estimate gene expression levels. Fisher’s exact test was used to calculate *p* values. We screened for differential genes with corrected *p* < 0.05 and a fold change >1.5.

### Whole-genome methylation sequencing

CD4^+^ T cells were sorted from splenocytes of WT, *Dnmt1*^flox/flox^, or *Dnmt1*^flox/flox^ *Cd4*^cre/+^ mice with or without *in vitro* stimulation. Cell samples were then sent to Shanghai OE Biotech Co. for low-input DNA library. We treated them with sodium bisulfite using the EZ DNA Methylation Gold Kit from Zymo Research and analyzed them using the Illumina DRAGEN Bio-IT platform. We used fastp for sample quality control, with the following filtering criteria: (1) filter out reads with a high-quality base (Q20) percentage below 70%; (2) remove low-quality bases with a quality value less than 20 at the 3′ end; (3) remove reads containing bases other than A, T, C, and G. To obtain clean reads, we used Bismark for methylation data alignment analysis against the reference genome. We analyzed the alignment results from the Bismark software using the MethylKit software. We took the 2-kb region upstream of the gene transcription start site as the gene promoter region and compared the levels of promoter methylation modifications between different samples within this region. We used Fisher’s exact test model to analyze the differentially methylated promoters (DMPs) between groups. IGV was used for data visualization.

### *In vitro* T cell stimulation

CD4^+^ T cells were sorted from splenocytes of WT, *Dnmt1*^flox/flox^, or *Dnmt1*^flox/flox^ *Cd4*^cre/+^ mice using the Dynabeads Untouched Mouse CD4^+^ T Cells Kit. According to the manufacturer’s instructions, the Dynabeads Mouse T cell activator CD3/CD28 (Thermo Fisher Scientific, Waltham, MA) and recombined IL-2 (Abcam, Cambridge) were used to activate T cells *in vitro*, and the cells were analyzed 48 h later using flow cytometry.

### Methylation-specific PCR and bisulfite sequencing PCR

The EZ DNA Methylation Gold Kit from Zymo was used to prepare bisulfited DNA. CpG island sites in the CDKN1A promoter region (2 kb upstream of the start codon) were predicted using the MethHC database, and primers were designed accordingly. After PCR amplification of the DNA samples, the target bands were detected using agarose gel electrophoresis. The primers are listed in Table S5.

Target bands were excised from the gel. The plasmid pMD18-T vector (Takara, Kyoto, Japan) was used to transform the target band DNA into competent bacterial colonies, followed by screening for positive colonies with antibiotics. After PCR amplification of the bacterial culture, gel electrophoresis was performed on the products, and finally, the bands of the desired size were selected for sequencing. The PCR primers are listed in Table S6 as well as the full amplicon sequences with CpG sites highlighted in red.

### Construction of a stable si-DNMT1 human cell line

The lentivirus LV-DNMT1-RNAi(14369-1) used for silencing DNMT1 (titer: 2.0 × 10^9^ TU/mL) and the negative control lentivirus CON077 (titer: 6.0 × 10^8^ TU/mL) were purchased from Shanghai Genechem Co., Ltd. 293T cells in the logarithmic growth phase were digested with trypsin, prepared into a cell suspension of 1 × 10^5^ cells/mL using DMEM complete and seeded in a six well plate at 2 mL/well. Negative control lentivirus CON077 (3.33 μL) was added to the control group cells and 1 μL of lentivirus LV-DNMT1-RNAi(14369-1) was added to the knockdown group cells. The original medium was replaced with fresh culture medium after 16 h. After 72 h of infection, puromycin was used to select for positively transfected cells. Since the vector carries green fluorescent protein (GFP), the expression of GFP could be observed under a fluorescence microscope. Once the fluorescence rate reached 100%, we halved the amount of puromycin and continued to select and expand the infected cells while collecting cells to perform qPCR to detect the transcriptional level of the DNMT1 gene. The primers are listed in Table S7.

### The UNOS heart registry analysis

A retrospective cohort analysis was conducted on adult heart transplant recipients in the United States from January 2010 to December 2021 in the UNOS database. The study excluded patients with multiple organ transplants, patients who underwent re-transplantations, and those previously transplanted. Furthermore, patients without recorded follow-up graft status in the database were also excluded. The primary endpoint was graft rejection failure at the 5-year follow-up after transplantation, while the secondary outcome of interest was whether the patient required treatment for rejection during the first post-transplant year. The data used in this study were generously supplied by UNOS as the contractor of the Organ Procurement and Transplantation Network (OPTN). The interpretation and reporting of OPTN heart registry data are the responsibility of the authors and should not be considered an official policy or interpretation by the OPTN or the US government.

The multivariable Cox proportional hazards regression model was used to identify independent predictors of graft failure due to rejection. The model included the pre-specified donor risk score as a continuous variable.^44^ Results were reported as HR with 95% CI. The proportional hazards assumption was verified using Schoenfeld residuals. All statistical analyses were performed using R software (R Foundation for Statistical Computing) with a two-tailed significance level of 0.05.

### PBMC isolation in heart transplantation patients

This study complied with the Declaration of Helsinki and was approved by the Ethics Committee of Union Hospital and Tongji Medical College (UHCT241110). Informed consent was obtained from all patients and healthy control subjects. The study participants were divided into the following two groups: heart transplant recipients who have survived more than 1 year (heart transplantation group, HTx, *n* = 10) and an age- and gender-matched healthy control group (*n* = 10). The demographic information on HTx and controls is summarized in Table S1. Peripheral blood was collected from patients who underwent follow-up visits after heart transplantation. Mononuclear cells were isolated using Ficoll and used for subsequent testing. Patients with cancer and those with recent infections were excluded.

### scRNA-seq data processing and analysis

Public scRNA-seq datasets from cardiac transplant models were integrated from multiple public datasets (Chang et al.,^45^ *n* = 1 each group; GEO: GSE288048, control *n* = 2; GEO: GSE151048, iso-graft *n* = 2; GEO: GSE249989, allo-graft *n* = 1; GEO: GSE262851, allo-graft *n* = 1) encompassing nine samples across three experimental conditions: control (naive mice, *n* = 3), syngeneic grafts (iso-graft, post-operative day 6, *n* = 3), and allogeneic grafts (allo-graft, post-operative day 6, *n* = 3). Detailed information on the database and donors/recipients is provided in Table S8. Raw count matrices were processed using the Seurat (v.5.0.1) pipeline.^44^ Cells were filtered based on quality control metrics, retaining cells with >600 and <6,000 unique molecular identifiers (UMIs) and <10% mitochondrial gene content. Doublets were identified and removed using scDblFinder,^46^ and ambient RNA contamination was corrected using decontX.^47^ Poorly expressed genes and non-informative genes (mitochondrial, ribosomal, immunoglobulin, pseudogenes, and genes lacking Entrez ID annotations) were excluded from downstream analysis. Data integration was performed separately for the total immune compartment and T cell subsets using Harmony integration.^48^ Briefly, counts were normalized, 2,000 highly variable features were selected, data were scaled, and principal-component analysis (PCA) was performed. Harmony was then applied to integrate samples across different batches using the first 20 principal components.

Unsupervised clustering was performed on the integrated data using the FindNeighbors and FindClusters functions (resolution = 0.4) based on Harmony embeddings. Major immune lineages were annotated using established marker genes: myeloid cells (*Cd68* and *C1qb*), B cells (*Bank1* and *Cd19*), neutrophils (*S100a9* and *Csf3r*), DCs (*Itgax* and *Napsa*), NK cells (*Nkg7* and *Klrd1*), and lymphoid cells (*Cd3e* and *Cd247*).

For T cell-specific analysis, lymphoid clusters were subsetted and re-integrated with cell-cycle scoring and regression (S and G2M scores) to mitigate cell-cycle effects. Cell states were annotated using a combination of unsupervised clustering and projection onto a reference T cell atlas using ProjecTILs.^46^ CD4^+^ T cell subsets were manually annotated as naive (*Sell* and *Ccr7*), early activation (*Cd69*), Th1 (*Tbx21* and *Ccr5*), Tfh (*Bcl6* and *Cxcr5*), Th2 (*Gata3* and *Il5*), Treg (*Foxp3* and *Il2ra*), and ILCs (*Rorc* and *Il23r*). CD8^+^ T cell subsets were annotated as naive (*Sell* and *Ccr7*), early activated (*Fosl2* and *Stat4*), TEM (*Cd44*; *Havcr2* and *Irf8*), Tpex (*Tcf7* and *Slamf6*), TCM (*Nkg7* and *Cx3cr1*), and IFN stimulated (*Ifit1*, *Isg15*, and *Rasad2*).

Genes that were differentially expressed between conditions and cell states were identified using the FindMarkers function with a MAST test^49^ requiring an adjusted *p* < 0.05 and log2 fold-change >0.25. Gene Ontology biological process (GOBP) enrichment analysis was performed using clusterProfiler (v.4.0.0).^50^ Pre-defined gene signatures for T cell functional programs (e.g., activation/effector or cytotoxicity) were obtained from the literature and converted to mouse orthologs.^11^ Module scores for these signatures were calculated using the UCell algorithm.^51^

CD4^+^ (naive to Th1/Tfh/Th2/Treg) and CD8^+^ (naive to TEM) differentiation trajectories were constructed using Slingshot on the integrated uniform manifold approximation and projection (UMAP) embeddings.^52^ We defined naive T cells (high expression of Sell, Ccr7, and Lef1) as the root state, representing the starting point of the T cell differentiation continuum. Th1, Th2, Tfh, and Treg clusters were designated as terminal states on the basis of their distinct expression of lineage-defining transcription factors and their established roles as differentiated effector or regulatory CD4^+^ T cell lineages in the context of the alloresponse.^53^^,^^54^^,^^55^ As for CD8^+^ T cell lineages, TEMs were designated as the terminal state on the basis of their cytotoxic function in acute transplant rejection.^56^ Genes dynamically expressed along pseudotime were identified using TradeSeq with generalized additive models (7 knots). Genes were considered markers if they exhibited a false discovery rate (FDR)-adjusted *p* < 0.05.^57^ The Mfuzz algorithm was used to cluster genes based on their expression patterns, and functional enrichment of co-expression modules was performed using GOBP and KEGG pathway analysis.

To quantify the effectiveness of batch effect removal and the preservation of biological heterogeneity across the nine integrated samples (*n* = 9, from five technical batches), we employed the Local Inverse Simpson’s Index (LISI). LISI scores were calculated using the lisi R package (v.1.0) based on the first 30 Harmony-integrated dimensions. For each cell, the local neighborhood (k = 30) was evaluated to determine the number of distinct datasets represented. An integration LISI (iLISI) score was assigned, where a score of 1 indicates that the neighborhood consists of cells from a single batch (poor mixing), while a score approaching 5 (the total number of technical batches) indicates that technical replicates are well intermingled within the same cluster.

### GEO database analysis

For the sequencing data of human samples, the R package Bioconductor was used to perform probe conversion on the whole-blood RNA-seq results of heart transplant recipients without rejection from GSE56967 and healthy controls from GSE14882. The FRIDMAN senescence-related gene set was loaded into the MSig database and compared with the sequencing results. The heatmap showed the relative FPKM of each gene, with CD3E as the reference for relative expression. The R package ggplot2 was used to create heatmaps.

### Statistical analysis

Data are expressed as mean ± standard deviation and analyzed with Prism version 8.0 (GraphPad Software). Graft survival was plotted by the Kaplan-Meier method, and analysis was performed using the log rank test. The other measurements were performed using unpaired Student’s *t* test. The sample size chosen for the animal experiments was estimated based on the literature and our previous experience. A *p* < 0.05 was considered significant. The statistical significance in the figures is indicated as follows: not significant (ns), *p* > 0.05; ∗*p* < 0.05, ∗∗*p* < 0.01, and ∗∗∗*p* < 0.001.

### Data and code availability

Raw sequencing data have been deposited in the NCBI Sequence Read Archive under BioProject PRJNA1293696.

## Acknowledgments

This work was supported by the 10.13039/501100001809National Natural Science Foundation of China (82470423 and 82500494), the 10.13039/501100012166National Key Research and Development Program (2021YFA1101900), and the 10.13039/501100003819Hubei Provincial Natural Science Foundation Projects (JCZRYB202500142). We thank LetPub (www.letpub.com.cn) for its linguistic assistance during the preparation of the manuscript.

## Author contributions

This study was conceived and designed by Y.C. and Y.W. Y.C. curated the data. Formal analysis was performed by W.Y.Y. and C.L. Funding was acquired by N.D. and Y.W. Experiments were carried out by Y.C., C.L., Z.W., and J.H. Methodology was developed by Y.C. and C.L. Project administration was handled by Y.W. and Z.L. Resources were provided by N.D. and Y.W. Software was developed and maintained by W.Y.Y. Supervision was provided by Y.W., F.L., and Z.L. Validation was conducted by Y.W., W.Y.Y., and Z.L. Data visualization was created by W.Y.Y. and Y.P. The original draft was written by Y.C., W.Y.Y., and C.L. The final manuscript was reviewed and edited by Y.W., Z.L., and N.D.

## Declaration of interests

The authors declare there is no conflict of interest.

## Supplemental information

Document S1. Figures S1–S7 and Tables S1–S8

Document S2. Article plus supplemental information
