# Supplementary material for: T cell senescence induced by DNMT1 deletion mediates cardiac transplant tolerance via altered CDKN1A methylation
Source: Mol Ther. 2026 Feb 13;34(6):3484–502. doi: 10.1016/j.ymthe.2026.02.015 (PMC13239813; doi:10.1016/j.ymthe.2026.02.015)
Supplement: Supplementary file 1 [file mmc1.pdf]

YMTHE, Volume *34*

Supplemental Information

T cell senescence induced by DNMT1 deletion mediates cardiac transplant tolerance via altered CDKN1A methylation

Yuqi Chen, Wai Yen Yim, Chenghao Li, Zihao Wang, Jincheng Hou, Yongbu Peng, Fayuan Liu, Zongtao Liu, Nianguo Dong, and Yixuan Wang

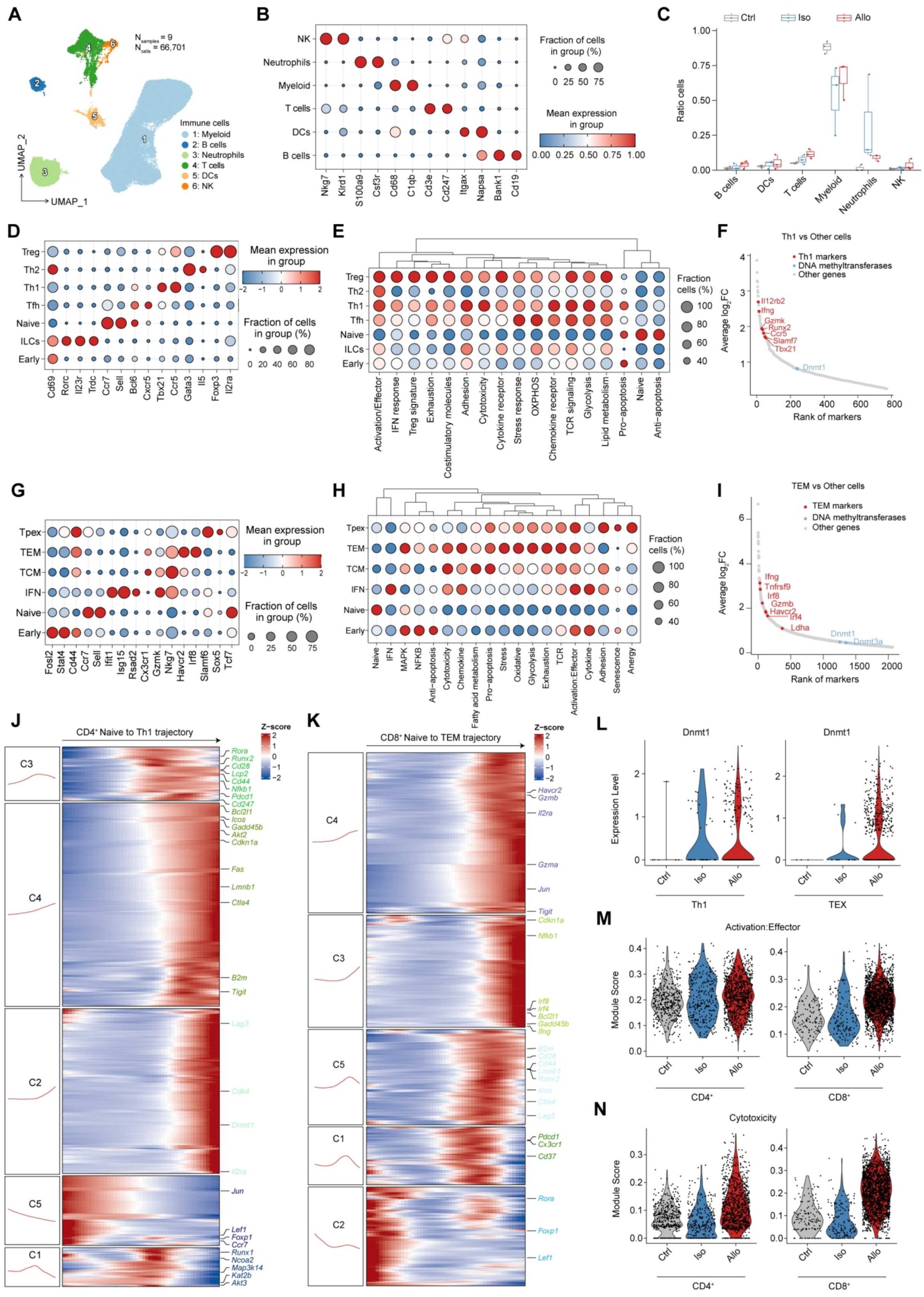

A
B
C
D
E
F
G
H
I
J
K
L
M
N
UMAP_1
UMAP_2
Immune cells
1: Myeloid
2: B cells
3: Neutrophils
4: T cells
5: DCs
6: NK
NK
Neutrophils
Myeloid
T cells
DCs
B cells
Nkg7
Klrd1
S100a9
Csf3r
Cd68
C1qb
Cd3e
Cd247
Itgax
Napsa
Bank1
Cd19
Fraction of cells in group (%)
Mean expression in group
Ctrl
Iso
Allo
Ratio cells
Treg
Th2
Th1
Tfh
Naive
ILCs
Early
Cd69
Rorc
Il23r
Trdc
Ccr7
Sell
Bcl6
Cxcr5
Tbx21
Ccr5
Gata3
Il5
Foxp3
Il2ra
Activation/Effector
IFN response
Treg signature
Exhaustion
Costimulatory molecules
Adhesion
Cytotoxicity
Cytokine receptor
Stress response
OXPHOS
Chemokine receptor
TCR signaling
Glycolysis
Lipid metabolism
Pro-apoptosis
Anti-apoptosis
Fraction cells (%)
Th1 vs Other cells
Th1 markers
DNA methyltransferases
Other genes
Il12rb2
Ifng
Gzmk
Runx2
Ccr5
Slamf7
Tbx21
Dnmt1
Average log2FC
Rank of markers
Tpex
TEM
TCM
IFN
Fosl2
Stat4
Cd44
Ifit1
Isg15
Rsad2
Cx3cr1
Nkg7
Havcr2
Irf8
Slamf6
Sox5
Tcf7
MAPK
NFKB
Chemokine
Fatty acid metabolism
Stress
Oxidative
TCR
Activation:Effector
Cytokine
Senescence
Anergy
TEM vs Other cells
TEM markers
Tnfrsf9
Gzmb
Irf4
Ldha
Dnmt3a
CD4+ Naive to Th1 trajectory
Z-score
C3
C4
C2
C5
C1
Rora
Cd28
Lcp2
Nfkb1
Pdcd1
Bcl2l1
Icos
Gadd45b
Akt2
Cdkn1a
Fas
Lmnb1
Ctla4
B2m
Tigit
Lag3
Cdk4
Il2ra
Jun
Lef1
Foxp1
Runx1
Ncoa2
Map3k14
Kat2b
Akt3
CD8+ Naive to TEM trajectory
Gzma
Cd37
Dnmt1
Expression Level
Th1
TEX
Module Score
CD4+
CD8+
Cytotoxicity

Figure S1. (A) UMAP of integrated immune cells from nine samples across three experimental groups: Control, syngeneic graft (Iso-graft, post-operative day 6), and allogeneic graft (Allo-graft, post-operative day 6), total N=3 in each group. Data were integrated from multiple public datasets (Chang et al.[45], N=1 each group; GEO: GSE288048, Control N=2; GSE151048, Iso-graft N=2; GSE249989, Allo-graft N=1; GSE262851, Allo-graft N=1). Cells are colored by major immune lineage: myeloid cells, B cells, neutrophils, lymphoid cells, dendritic cells (DCs), and natural killer (NK) cells. (B) Dot plot of defining marker genes for the major immune lineages. Dot color represents the mean scaled expression level, and dot size indicates the percentage of cells within a cluster expressing the gene. (C) Proportional abundance of each major immune lineage across the three experimental groups. Each of the nine dots corresponds to one independent biological sample (n=3 for Ctrl, n=3 for Isograft, n=3 for Allograft). Box plots depict the median and interquartile range.Statistical significance was determined by a non-parametric Wilcoxon rank-sum test. (D) Dot plot of selected marker genes defining the $CD4^+$ T cell states. The color scale represents the mean scaled expression level (red, high; blue, low), and dot size corresponds to the percentage of cells within a cluster expressing the gene.(E) Enrichment scores for curated gene signatures representing key $CD4^+$ T cell functional programs. (F) Ranked list of upregulated differentially expressed genes (adjusted p-value < 0.05) in Th1 cells compared to all other $CD4^+$ T cell states. The Y-axis shows the average log2 fold-change (Log2FC). Th1-defining genes are highlighted in red, and DNA methyltransferase-related genes are highlighted in blue. (G) Dot plot of selected marker genes defining the $CD8^+$ T cell states. The color scale represents the mean scaled expression level (red, high; blue, low), and dot size corresponds to the percentage of cells within a cluster expressing the gene. (H) Enrichment scores for curated gene signatures representing key $CD8^+$ T cell functional programs.(I) Ranked list of upregulated differentially expressed genes (adjusted p-value < 0.05) in TEM cells compared to all other $CD8^+$ T cell states. The Y-axis shows the average log2 fold-change (Log2FC). TEM-defining genes are highlighted in red, and DNA methyltransferase-related genes are highlighted in blue. (J) Clustered heatmap generated by the Mfuzz algorithm, depicting the co-expression patterns of genes differentially expressed along the $CD4^+$ Naïve to T helper 1 (Th1) trajectory (K) Parallel analysis for the $CD8^+$ Naïve to T effector memory (TEM) differentiation trajectory, showing gene co-expression patterns. (L) Violin plots illustrating the expression levels of *Dnmt1* within Th1 (left) and TEM (right) cell populations across the experimental groups.(M) Module scores for an 'Activation:Effector' gene signature in $CD4^+$ (left) and $CD8^+$ (right) T cells across groups. (N) Module scores for a 'Cytotoxicity' gene signature in $CD4^+$ (left) and $CD8^+$ (right) T cells across groups.

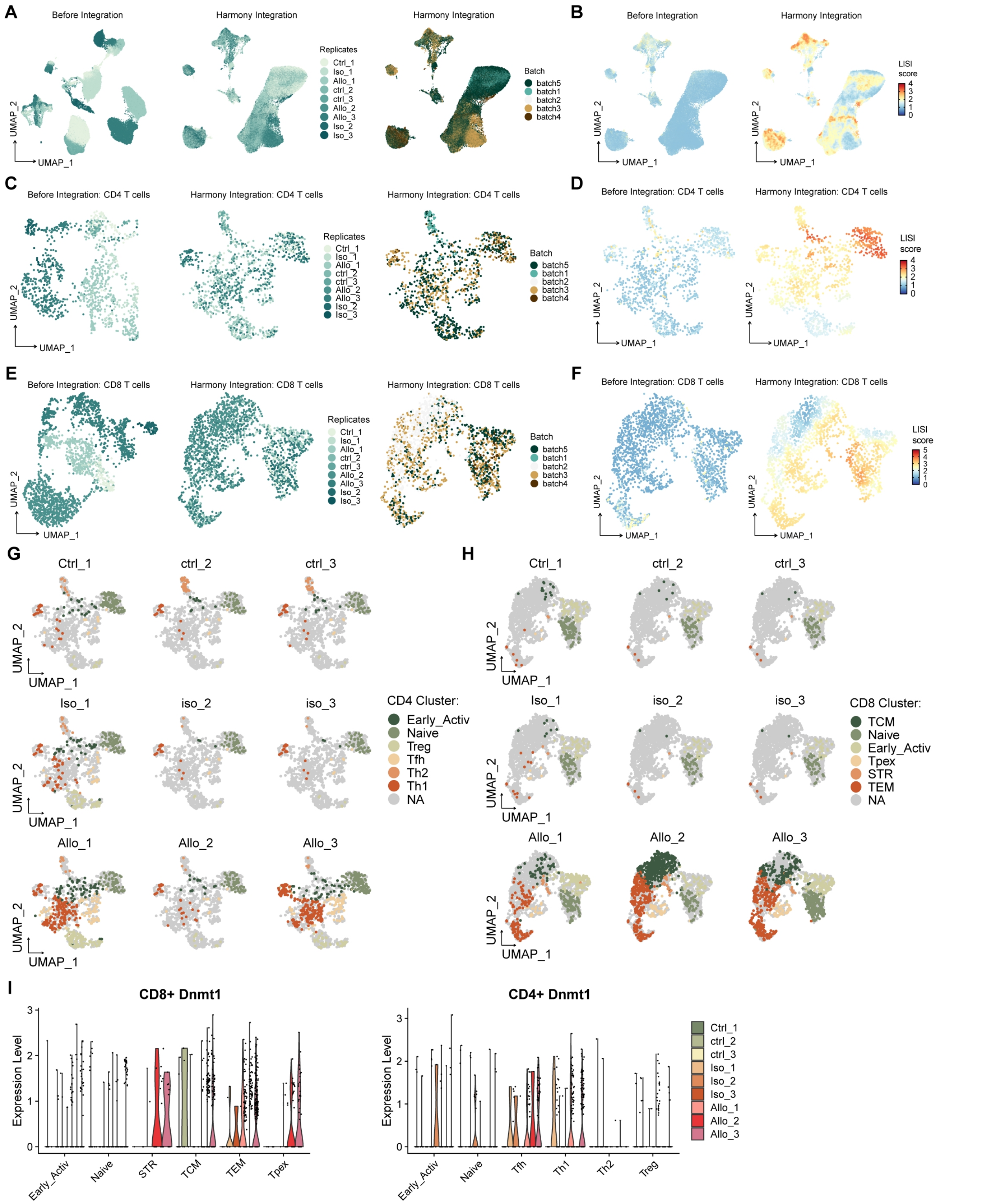

Figure S2. Validation of scRNA-seq data integration and batch effect removal. (A, C, E) UMAP projections showing the integration of nine samples (from five technical batches) before and after Harmony integration for (A) total cells, (B) CD4^{+} T cells, and (C) CD8^{+} T cells. Cells are colored by biological replicate (middle panels) and technical batch (right panels) to demonstrate the transition from

batch-specific clustering to integrated mixing. (B, D, F) Quantitative assessment of batch mixing using Local Inverse Simpson's Index (LISI) scores for (B) total cells, (D) CD4$^+$ T cells, and (F) CD8$^+$ T cells. High LISI scores (yellow/red) post-integration indicate that multiple batches are represented within local cellular neighborhoods, confirming successful integration. (G, H) Individual sample UMAP plots showing the distribution of identified (G) CD4$^+$ T cell clusters and (H) CD8$^+$ T cell clusters across all nine replicates (Ctrl_1-3, Iso_1-3, and Allo_1-3). The consistent presence and distribution of key biological states (e.g., Naive, Th1, TEM) across replicates within the same experimental group demonstrate that the biological signal is robust and independent of technical heterogeneity. (I) Violin plot of Dnmt1 expression across identified T-cell subsets for each individual sample.

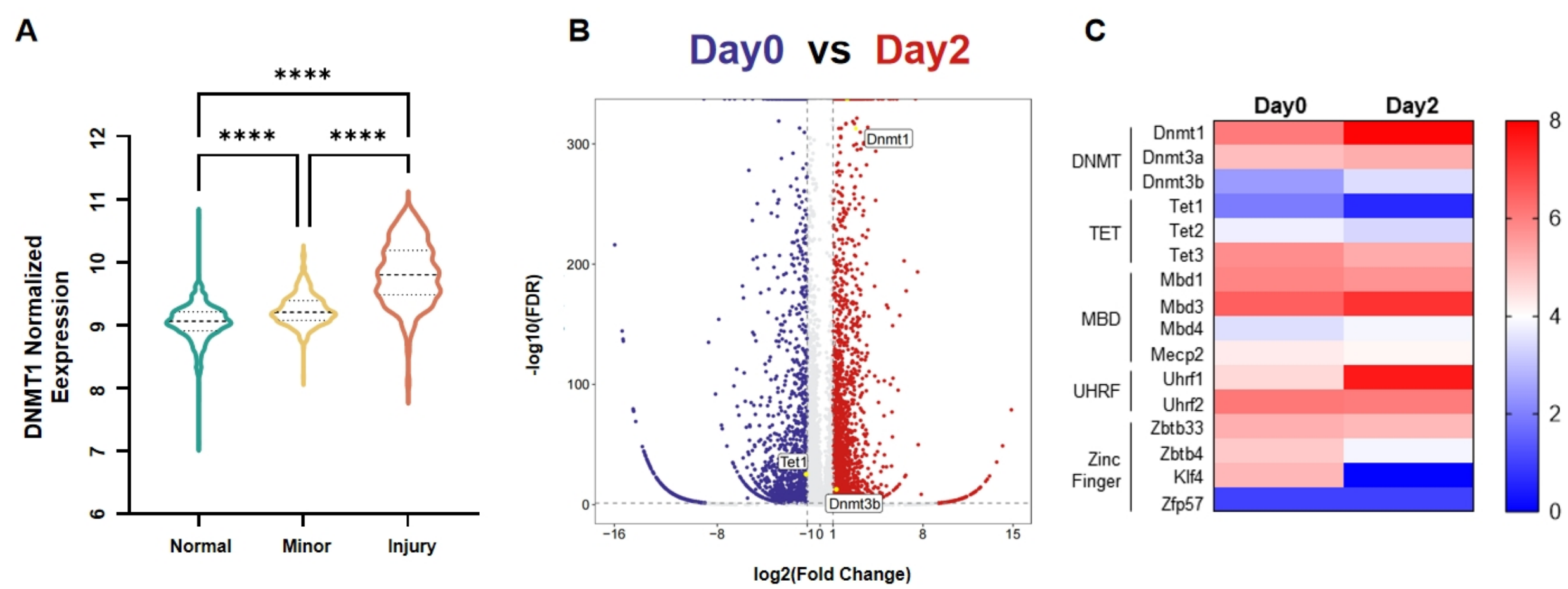

Figure S3. (A) Violin plot of DNMT1 expression of endomyocardial biopsy tissue in different grades of T cell-mediated rejection in the GSE150059 dataset. Following the method described by the original depositor, we divided the samples into three groups.Normal(n=462); Minor(n=359); Injury(n=146). Injury = (Early injury + Potential TCMR + TCMR). (B) Volcano plot of differentially expressed genes in CD4$^+$ T cells on the day 2 and day 0 of in vitro activation. (C) Heatmap of DNA methylation-related genes in CD4$^+$ T cells on the day 2 and day 0 of in vitro activation.

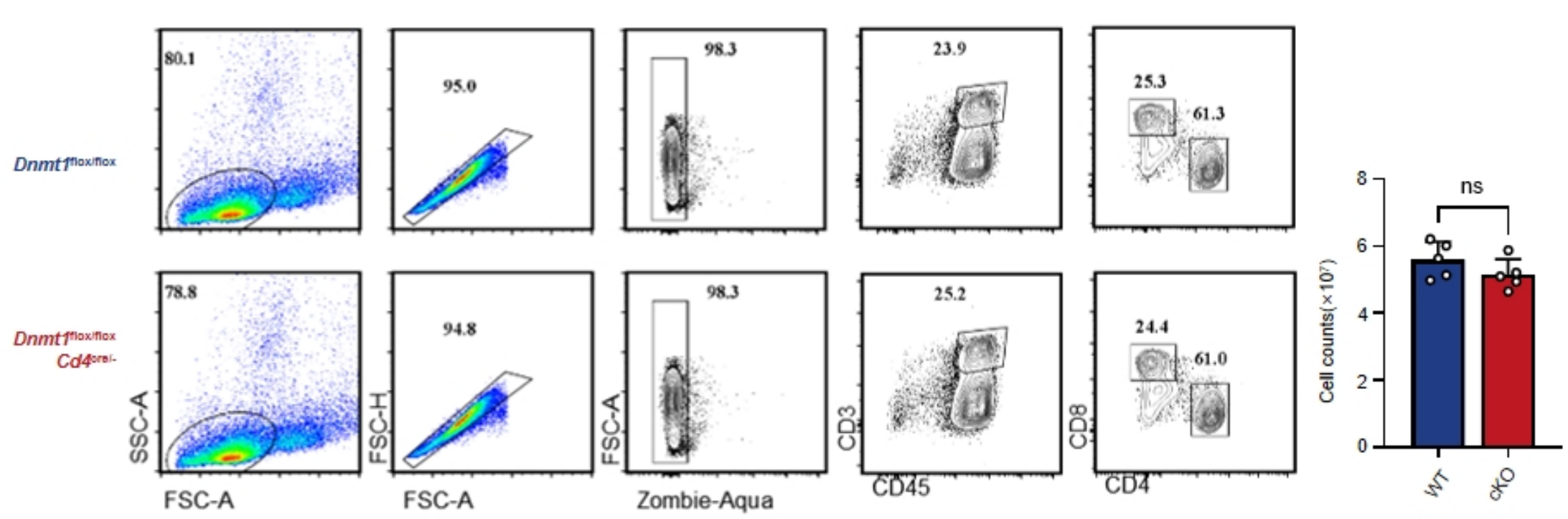

Figure S4. Gating strategy for flow cytometry and the proportion and count of T cell subsets in *Dnmt1*$^{flox/flox}$ *Cd4*$^{cre/+}$ and *Dnmt1*$^{flox/flox}$ control mice spleens before cardiac transplantation. Ns, no significance; Unpaired Student's t test.

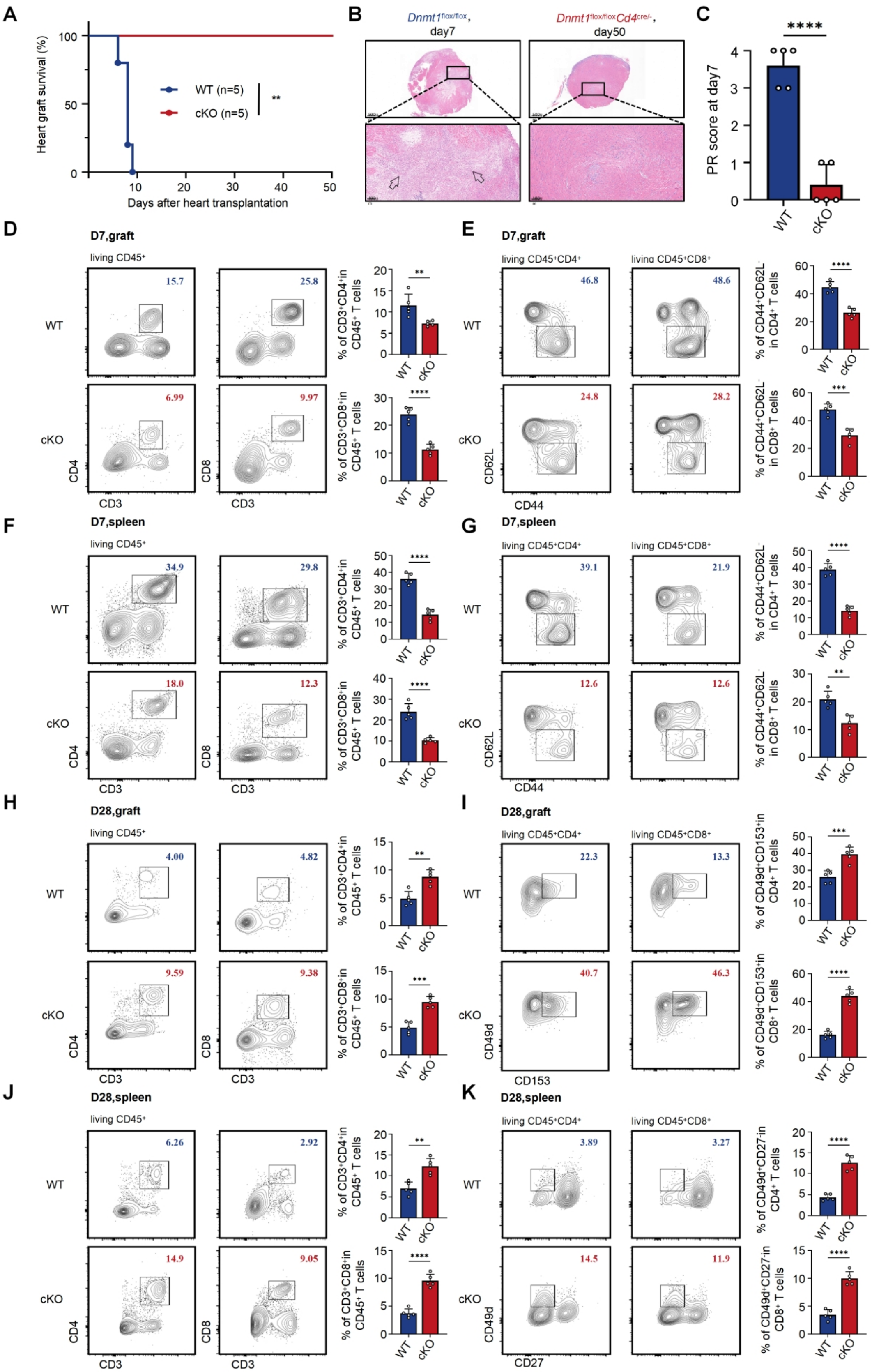
A
Heart graft survival (%)
Days after heart transplantation
WT (n=5)
cKO (n=5)
**
B
Dnmt1flox/flox,
day7
Dnmt1flox/floxCd4cre/-,
day50
C
****
PR score at day7
WT
cKO
D
D7,graft
living CD45+
15.7
25.8
6.99
9.97
% of CD3+CD4+in CD45+ T cells
% of CD3+CD8+in CD45+ T cells
CD4
CD8
CD3
E
D7,graft
living CD45+CD4+
living CD45+CD8+
46.8
48.6
24.8
28.2
% of CD44+CD62L- in CD4+ T cells
% of CD44+CD62L- in CD8+ T cells
CD62L
CD44
F
D7,spleen
living CD45+
34.9
29.8
18.0
12.3
G
D7,spleen
39.1
21.9
12.6
12.6
H
D28,graft
4.00
4.82
9.59
9.38
I
D28,graft
22.3
13.3
40.7
46.3
% of CD49d+CD153+in CD4+ T cells
% of CD49d+CD153+in CD8+ T cells
CD49d
CD153
J
D28,spleen
6.26
2.92
14.9
9.05
K
D28,spleen
3.89
3.27
14.5
11.9
% of CD49d+CD27-in CD4+ T cells
% of CD49d+CD27-in CD8+ T cells
CD27

Figure S5. Related experimental results in female mice after transplantation. (A) Percentage of heart and skin allograft survival after transplantation.**, *p* < 0.01;log-rank test. (B) Representative images of H&E staining of grafts from *Dnmt1*$^{flox/flox}$ *Cd4*$^{cre/+}$ and control mice after cardiac transplantation. ×50 and ×200 magnification. (C) Bar graphs of PR score at day 7 after cardiac transplantation. n=5, ****, *p* < 0.0001; Unpaired Student's t test. (D) Representative contour plots and bar graphs showing the % of CD4$^+$ and CD8$^+$ T cells in CD45$^+$ cells of grafts from *Dnmt1*$^{flox/flox}$ *Cd4*$^{cre/+}$ and *Dnmt1*$^{flox/flox}$ control mice at day 7 after cardiac transplantation. ns, no significance; **, *p* < 0.01;****, *p* < 0.0001; unpaired Student's t- test. (E) Representative contour plots and bar graphs showing the % of CD44$^+$CD62L$^-$ cells in CD4$^+$ and CD8$^+$ T cells of grafts from *Dnmt1*$^{flox/flox}$ *Cd4*$^{cre/+}$ and *Dnmt1*$^{flox/flox}$ control mice at day 7 after cardiac transplantation. ***, *p* < 0.001; ****, *p* < 0.0001; Unpaired Student's t test. (F) Representative contour plots and bar graphs showing the % of CD4$^+$ and CD8$^+$ T cells in CD45$^+$ cells of spleens from *Dnmt1*$^{flox/flox}$ *Cd4*$^{cre/+}$ and *Dnmt1*$^{flox/flox}$ control mice at day 7 after cardiac transplantation. ns, no significance; ****, *p* < 0.0001; unpaired Student's t- test. (G) Representative contour plots and bar graphs showing the % of CD44$^+$CD62L$^-$ cells in CD4$^+$ and CD8$^+$ T cells of spleens from *Dnmt1*$^{flox/flox}$ *Cd4*$^{cre/+}$ and *Dnmt1*$^{flox/flox}$ control mice at day 7 after cardiac transplantation. **, *p* < 0.01; ****, *p* < 0.0001; Unpaired Student's t test.) (H) Representative contour plots and bar graphs showing the % of CD4$^+$ and CD8$^+$ T cells in CD45$^+$ cells of grafts from *Dnmt1*$^{flox/flox}$ *Cd4*$^{cre/+}$ and *Dnmt1*$^{flox/flox}$ control mice at day 28 after cardiac transplantation. **, *p* < 0.01; ***, *p* < 0.001; Unpaired Student's t test. (I) Representative contour plots and bar graphs showing the % of CD49d$^+$CD153$^+$ T cells in CD4$^+$ and CD8$^+$ T cells of grafts from *Dnmt1*$^{flox/flox}$ *Cd4*$^{cre/+}$ and *Dnmt1*$^{flox/flox}$ control mice at day 28 after cardiac transplantation. ***, *p* < 0.001; ****, *p* < 0.0001; Unpaired Student's t test. (J) Representative contour plots and bar graphs showing the % of CD4$^+$ and CD8$^+$ T cells in CD45$^+$ cells of spleens from *Dnmt1*$^{flox/flox}$ *Cd4*$^{cre/+}$ and *Dnmt1*$^{flox/flox}$ control mice at day 28 after cardiac transplantation. **, *p* < 0.01; ****, *p* < 0.0001; Unpaired Student's t test. (K) Representative contour plots and bar graphs showing the % of CD49d$^+$CD27$^-$ T cells in CD4$^+$ and CD8$^+$ T cells of spleens from *Dnmt1*$^{flox/flox}$ *Cd4*$^{cre/+}$ and *Dnmt1*$^{flox/flox}$ control mice at day 28 after cardiac transplantation. ****, *p* < 0.0001; Unpaired Student's t test.

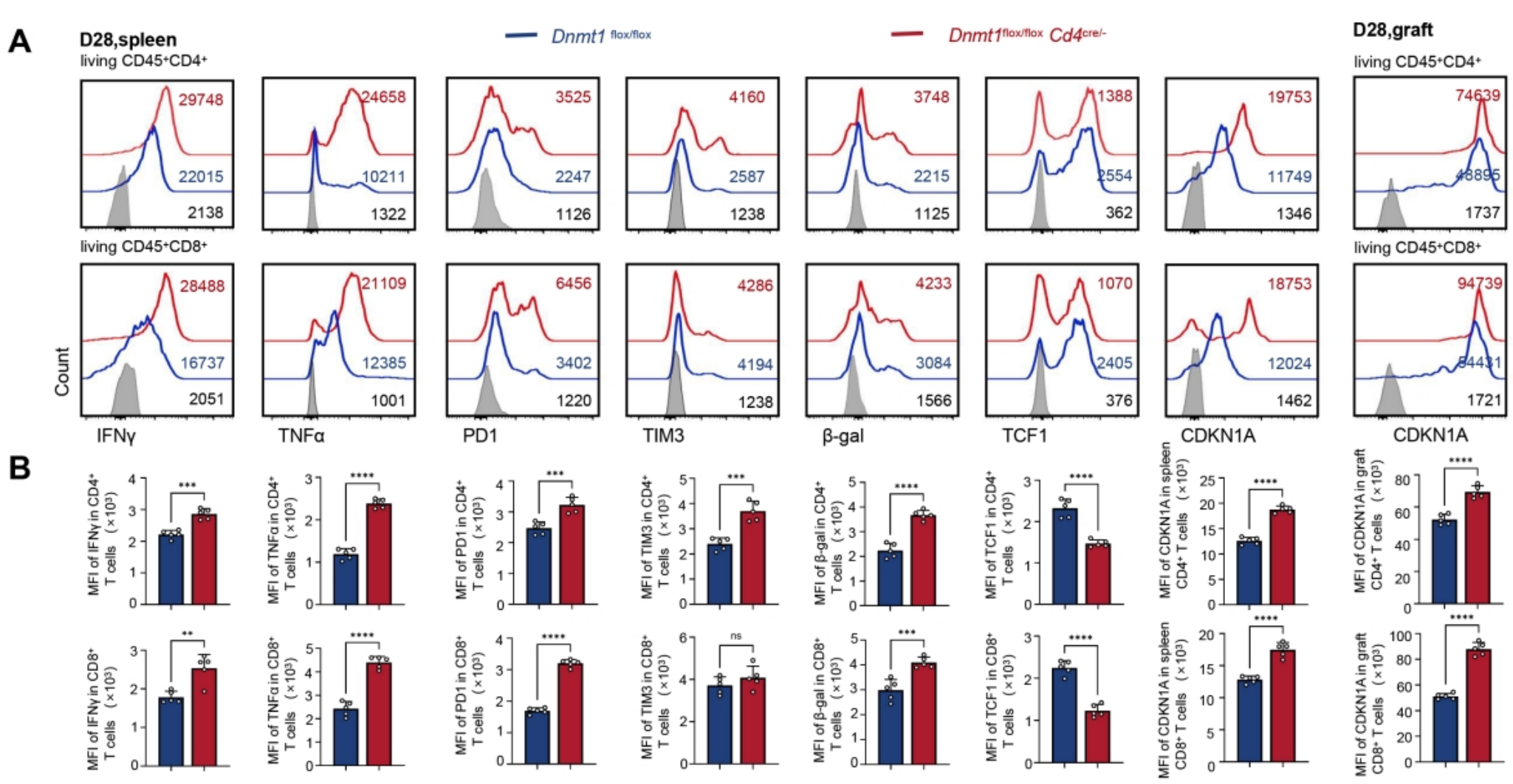

Figure S6. Representative histogram overlay and MFI bar graphs of TNFα, IFNγ, PD1, TIM3, TCF1, β-Gal and CDKN1A from spleens $CD4^+$ and $CD8^+$ T cells of *Dnmt1*$^{flox/flox}$ *Cd4*$^{cre/+}$ and *Dnmt1*$^{flox/flox}$ control mice at day 28 after cardiac transplantation. ***, $p < 0.001$; ****, $p < 0.0001$; Unpaired Student's t test.

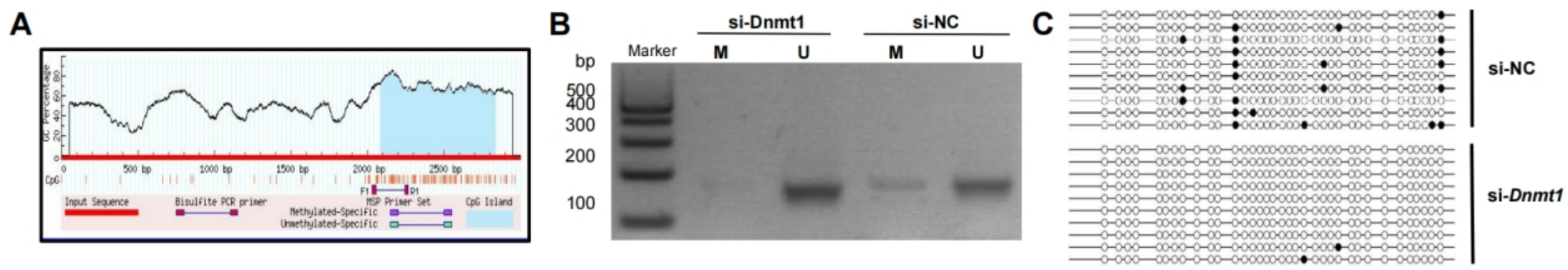

Figure S7. (I) Schematic diagram of CpG island prediction of CDKN1A promoter and methylation primer design of 293T cells. (J) Electrophoresis result image of methylation-specific PCR of si-DNMT1 and control 293T cells (K) Dot plot of CDKN1A promoter methylation levels obtained by BSP.

Table S1. Detailed p-values and effect sizes for single-cell statistical analysis

CellType	Subtype	group1	group2	n1	n2	statistic	p	effsize
All	Bcells	CONT	ISO	3	3	2	0.4	-0.5808402
All	Bcells	CONT	ALLO	3	3	2	0.4	-1.1635449
All	Bcells	ISO	ALLO	3	3	4	1	-0.6055981
All	DCs	CONT	ISO	3	3	3	0.7	-0.6703248
All	DCs	CONT	ALLO	3	3	3	0.7	-0.7020386
All	DCs	ISO	ALLO	3	3	4	1	-0.2824421
All	Lymphoid	CONT	ISO	3	3	3	0.7	-0.9711534
All	Lymphoid	CONT	ALLO	3	3	0	0.1	-2.6657657
All	Lymphoid	ISO	ALLO	3	3	1	0.2	-1.2071675
All	Myeloid	CONT	ISO	3	3	9	0.1	1.9458407
All	Myeloid	CONT	ALLO	3	3	9	0.1	2.1487671
All	Myeloid	ISO	ALLO	3	3	2	0.4	-0.646068
All	Neutrophils	CONT	ISO	3	3	0	0.076	-1.3072228
All	Neutrophils	CONT	ALLO	3	3	0	0.076	-3.2941884
All	Neutrophils	ISO	ALLO	3	3	9	0.1	0.9687226
All	NK	CONT	ISO	3	3	3	0.7	-0.336191
All	NK	CONT	ALLO	3	3	1	0.2	-0.9391453
All	NK	ISO	ALLO	3	3	3	0.7	-0.8064581
CD4	Early	CONT	ISO	3	3	5.5	0.747	0.09362662
CD4	Early	CONT	ALLO	3	3	1.5	0.134	-1.2360124
CD4	Early	ISO	ALLO	3	3	2	0.2	-1.00834628
CD4	ILCs	CONT	ISO	3	3	9	1	4.79949377
CD4	ILCs	CONT	ALLO	3	3	9	1	4.51271067
CD4	ILCs	ISO	ALLO	3	3	4	0.5	-0.14085249
CD4	Naïve	CONT	ISO	3	3	1	0.1	-1.20247011
CD4	Naïve	CONT	ALLO	3	3	3.5	0.412	-0.30271282
CD4	Naïve	ISO	ALLO	3	3	7	0.9	1.07544229
CD4	Tfh	CONT	ISO	3	3	0	0.038	-3.63579253
CD4	Tfh	CONT	ALLO	3	3	0	0.05	-2.66031772
CD4	Tfh	ISO	ALLO	3	3	2	0.188	-1.11044865
CD4	Th1	CONT	ISO	3	3	0	0.038	-3.15250568
CD4	Th1	CONT	ALLO	3	3	0	0.038	-7.74251099
CD4	Th1	ISO	ALLO	3	3	1	0.1	-1.59279064
CD4	Th2	CONT	ISO	3	3	7	0.9	1.52742813
CD4	Th2	CONT	ALLO	3	3	9	0.987	1.81916555
CD4	Th2	ISO	ALLO	3	3	6	0.812	0.72305958
CD4	Treg	CONT	ISO	3	3	1	0.092	-1.4250307
CD4	Treg	CONT	ALLO	3	3	0	0.038	-1.9411708
CD4	Treg	ISO	ALLO	3	3	4.5	0.588	-0.09419824
CD8	Early_Activ	CONT	ISO	3	3	0	0.05	-1.87231692

CD8	Early_Activ	CONT	ALLO	3	3	4	0.5	-0.032328031
CD8	Early_Activ	ISO	ALLO	3	3	9	1	1.801908428
CD8	Naive	CONT	ISO	3	3	6	0.8	0.767580314
CD8	Naive	CONT	ALLO	3	3	9	1	3.389209354
CD8	Naive	ISO	ALLO	3	3	9	1	2.036620291
CD8	STR	CONT	ISO	3	3	2	0.177	-1.404846467
CD8	STR	CONT	ALLO	3	3	0	0.038	-2.968862718
CD8	STR	ISO	ALLO	3	3	5	0.65	-0.002805805
CD8	TCM	CONT	ISO	3	3	8	0.95	1.636932764
CD8	TCM	CONT	ALLO	3	3	4	0.5	-0.167997273
CD8	TCM	ISO	ALLO	3	3	0	0.05	-1.405475514
CD8	TEM	CONT	ISO	3	3	1	0.092	-1.692964512
CD8	TEM	CONT	ALLO	3	3	0	0.038	-17.64906938
CD8	TEM	ISO	ALLO	3	3	0	0.05	-4.700241096
CD8	Tpex	CONT	ISO	3	3	3	0.321	-0.817230094
CD8	Tpex	CONT	ALLO	3	3	0	0.038	-5.563812984
CD8	Tpex	ISO	ALLO	3	3	2	0.2	-1.22556017

Table S2. The baseline characteristic of patients with cardiac transplantation and control patients.

	**Control, N = 10**	**HTx, N = 10**	***p*-value**
**Age**	51.5 (30.0-60.3)	58.0 (39.5-69.8)	0.583
**Gender, male (%)**	5 (50)	8 (80)	0.160
**BMI**	23.7 (23.0-27.0)	25.0 (23.7-28.7)	0.281
**Diagnosis, n (%)**	ASD，9 (90)<br>PDA，1 (10)	DCM, 7 (70)<br>ICM, 3 (30)	
**Diabetes, n (%)**	1 (10)	2 (20)	0.531
**Hypertension, n (%)**	1 (10)	1 (10)	1.000

ASD, atrial septal defect; PDA, patent ductus arteriosus; DCM, dilated cardiomyopathy; ICM, ischemic cardiomyopathy. Continuous variables are presented as mean ± standard deviation and compared using student-t test. Categorical variables are reported as frequencies and percentages and compared using the chi-square test.

Table S3. The baseline characteristic of donors and recipients in UNOS database

	**young, N = 3,051**	**mid, N = 13,516**	**old, N = 4,206**	***p*-value**
**Recipient age**	31 (25-36)	56 (50-60)	67 (66-69)	<0.001
**Recipient gender, male（%）**	1,923 (63)	10,020 (74)	3,401 (81)	<0.001
**Recipient BMI**	26.5	27.7 (24.4-31.4)	26.5 (23.8-29.7)	<0.001

	(22.5-31.2)			
**Donor age**	29 (23-36)	32 (24-41)	33 (25-44)	<0.001
**Donor gender, male（%）**	2,055 (67)	9,532 (71)	2,906 (69)	0.002
**Donor BMI**	26.0 (23.0-30.1)	26.7 (23.6-30.8)	26.6 (23.6-30.5)	<0.001
**Donor diabetes，n (%)**	86 (2.8)	512 (3.8)	205 (4.9)	<0.001
**Donor alcohol，n (%)**	463 (16)	2,360 (18)	807 (20)	<0.001
**Donor cigarette，n (%)**	268 (8.9)	1,677 (13)	629 (15)	<0.001
**ECMO，n (%)**	81 (2.7)	151 (1.1)	31 (0.7)	<0.001
**IABP，n (%)**	277 (9.1)	1,293 (9.6)	441 (10)	0.1

Continuous variables are presented as mean ± standard deviation and compared using one-way ANOVA. Categorical variables are reported as frequencies and percentages and compared using the chi-square test.

Table S4. Multivariate COX Regress Analysis of Graft Failure due to Rejection

**Characteristic**	**HR**	**95% CI**	**p-value**
Age Group			
Mid (Reference)	—	—	
Old	0.67	0.49, 0.91	0.010
Young	2.69	2.20, 3.29	<0.001
Donor Risk Score	0.98	0.93, 1.02	0.3
Total Bilirubin (mg/DL)	1.03	0.97, 1.09	0.3
Gender (Female vs Male)	0.82	0.67, 0.99	0.044
Creatinine (mg/DL)	1.06	0.96, 1.16	0.3
Dialysis at Listing (Yes vs. No)	1.58	0.89, 2.80	0.12
Pre-OP ECMO (Yes vs. No)	1.14	0.52, 2.51	0.7
Pre-OP IABP (Yes vs. No)	1.39	1.05, 1.86	0.023
Pre-OP Ventilator (Yes vs. No)	0.94	0.40, 2.20	0.9

CI, Confidence Interval; HR, Hazard Ratio; ECMO, Extracorporeal Membrane Oxygenation; IABP, Intra-Aortic Balloon Pump.Donor Risk Score was calculated as the sum of the following components:

ischemia time score (ISCHTIME <2 h=1, 2-4 h=2, 4-6 h=3, 6-8 h=4, ≥8 h=5) + donor age score (<40 years=0, 40-49 years=3, ≥50 years=5) + race mismatch score (match=0, mismatch=2) + BUN/creatinine ratio score (<30=0, ≥30=2).

Table S5. The primers of Methylation-specific PCR

M-Cdkn1a(M)	Forward	5'-GGTTAGGTTTAGTTGGTTCGGC-3'
	Reverse	5'-CCTCAACTAACGCAACTCAACG-3'
M-Cdkn1a (U)	Forward	5'- TTTGGTTAGGTTTAGTTGGTTTGGT-3'
	Reverse	5'- CACACCTCAACTAACACAACTCAACA-3'
H-Cdkn1a (M)	Forward	5'-GTTTTGTATACGAGTATTGAATTTGAGATC-3'
	Reverse	5'-TAAAACAAAAAAATAAAAAATTCGACG-3'
H-Cdkn1a (U)	Forward	5'- GTTTTGTATATGAGTATTGAATTTGAGATTG-3'
	Reverse	5'- TCTAAAACAAAAAAATAAAAAATTCAACA-3'

Table S6. The primers of Bisulfite Sequencing PCR

M-Cdkn1a	Forward	5'- TGTTTTTTTAGTATTTAGTGTATGG -3'
	Reverse	5'- AATCCAATAATATAAATCTACCAC -3'
TGTTTTTTTAGTATTTAGTGTATGGAACGTGGGTGGGATTAAGTTAAGGTATAGTAGTTGTTTGT<br>GTTTTTTTTTTTTTTGTTTTGTATACGAGTATTGAATTTGAGATCGCGTATATGTTAGATAAGTGT<br>TTTTTTATTGAGTTGCGTTTTTTTCGCGTTTTTTTTTTTTTCGTTTAATTTTTGAGTTTACGTTTTAT<br>TTAGACGTAAGTTTTTTTTTAGTTTTTTAGAATTATTATTTAGGCGTCGAATTTTTTATTTTTTTGT<br>TTTAGATTTTTAGGTGGTTGGCGTGGTAGATTTATATTATTGGATT		
H-Cdkn1a	Forward	5'- TAGTGTTGTGTTTTTTTGGAGAGTG -3'
	Reverse	5'- CTCTCTCACCTCCTCTAAATACCTC -3'
TAGTGTTGTGTTTTTTTGGAGAGTGTTAATTTATTTTTTAAGTAAAAAAAGTTAGATTTGTGG<br>TTTATTTCGTGGGGAAATGTGTTTAGCGTATTAACGTAGGCGAGGGATTGGGGGAGGAGGG<br>AAGTGTTTTTTTGTAGTACGCGAGGTTTCGGGATCGGTTGGTTTGTTGGAATTCGGTTAGGT<br>TTAGTTGGTTCGGCGTTGGGTAGTTAGGAGTTTGGGTTTCGGGGAGGGCGGTTTCGGGCGG<br>CGCGGTGGGTCGAGCGCGGGTTTCGTTTTTTTGAGGCGGGTTCGGGCGGGGCGGTTGTATA<br>TTAGGGTCGCGTTGAGTTGCGTTAGTTGAGGTGTGAGTAGTTGTCGAAGTTAGTTTTTTGTG<br>GAGTCGGAGTTGGGCGCGGATTCGTCGAGGTATCGAGGTATTTAGAGGAGGTGAGAGAG		

Table S7. The primers of qPCR after lentiviral transfection

β-Actin	Forward	5’ - GCGTGACATTAAGGAGAAGC -3’
	Reverse	5’ - CCACGTCACACTTCATGATGG -3’
Dnmt1	Forward	5’ - GACCATCAGGCATTCTACC -3’
	Reverse	5’ - AGGCTTTACATTTCCCACA -3’

Table S8. Comprehensive overview of all integrated public datasets

Dataset	PMID	Source	Donor strain	Donor sex	Host strain	Host sex	Sample_Name	Sorted cells
Chang et. al.	34870762	https://figshare.com/s/11d8b3c99165c5fd8cd3	BALB/c	Male	-	-	Ctrl_1	CD45+
Chang et. al.	34870762	https://figshare.com/s/11d8b3c99165c5fd8cd3	BALB/c	Male	BALB/c	Male	Iso_1	CD45+
Chang et. al.	34870762	https://figshare.com/s/11d8b3c99165c5fd8cd3	BALB/c	Male	C57BL/6J	Male	Allo_1	CD45+
GSE208425	-	NCBI GEO	C57BL/6J	Male	-	-	Ctrl_2	CD45+
GSE208425	-	NCBI GEO	C57BL/6J	Male	-	-	Ctrl_3	CD45+
GSE151048	35222420	NCBI GEO	BALB/c	Male	BALB/c	Male	Iso_2	-
GSE151048	35222420	NCBI GEO	BALB/c	Male	BALB/c	Male	Iso_3	-
GSE262851	38889142	NCBI GEO	BALB/c	Male	C57BL/6J	Male	Allo_2	CD45+ / Pan-DC
GSE249989	38669317	NCBI GEO	BALB/c	Male	BALB/c	Male	Allo_3	CD45+
